# Alterations of the *Coxiella burnetii* Replicative Vacuole Membrane Integrity and Interplay with the Autophagy Pathway

**DOI:** 10.3389/fcimb.2017.00112

**Published:** 2017-04-24

**Authors:** María E. Mansilla Pareja, Antonino Bongiovanni, Frank Lafont, María I. Colombo

**Affiliations:** ^1^Laboratorio de Biología Celular y Molecular—Instituto de Histología y Embriología (IHEM), Universidad Nacional de Cuyo, CONICET, Facultad de Ciencias MédicasMendoza, Argentina; ^2^Cellular Microbiology and Physics of Infection Group—Center of Infection and Immunity of Lille, Centre National de la Recherche Scientifique, Institut Pasteur of Lille, UMR8204, Institut National de la Santé Et de la Recherche Médical U1019, Lille Regional Hospital, University Center, Universite LilleLille, France

**Keywords:** *Coxiella burnetii*, autophagy, galectins, vacuole membrane, damage detection

## Abstract

*Coxiella burnetii*, the etiologic agent of Q fever, is a Gram-negative obligate intracellular bacterium. It has been previously described that both the endocytic and autophagic pathways contribute to the *Coxiella* replicative vacuole (CRV) generation. Galectins are β-galactoside-binding lectins that accumulate in the cytosol before being secreted via a non-conventional secretory pathway. It has been shown that Galectin-3, -8, -9 monitor bacteria vacuolar rupture and endosomal and lysosomal loss of membrane integrity through binding of host glycans exposed in the cytoplasm after membrane damage. Using microinjection of fluorescence-coupled dextrans, a FRET assay, and galectins distribution, we demonstrate that *Coxiella* infection actually result in transient phagosomal/CRV membrane damage in a Dot/Icm-dependent manner. We also show the association of different adaptor molecules involved in autophagy and of LC3 to the limiting membrane of the CRV. Moreover, we show that upon autophagy inhibition, the proportion of CRVs labeled with galectins and less acidified increases which is associated with bacteria replication impairment. Based on these observations, we propose that autophagy can facilitate resealing of intracellular damaged membranes.

## Introduction

The obligate intracellular bacterium *Coxiella burnetii*, the etiologic agent of Q-fever (Voth and Heinzen, 2007), is a highly infectious human pathogen responsible for a global zoonotic disease called Q fever. It is found in a wide range of hosts, including livestock and humans. *C. burnetii* inhabits mainly monocytes/macrophages but it can also infect a wide variety of host cells *in vitro* (Voth and Heinzen, 2007). It directs the biogenesis of a large membrane-bound compartment called “*Coxiella* replicative vacuole” (CRV). This compartment has late endosome-lysosome characteristics. *Coxiella* transits the endosomal pathway as revealed by the presence of the Rab5 and EEA1 markers at early times of infection (Romano et al., 2007). Afterwards, the phagosome containing *C. burnetii* interacts with late endosomes and lysosomes as demonstrated by the recruitment of Rab7 (Berón et al., 2002) and the presence of molecules such as LAMP-1, CD63, and mannose-6-phosphate receptor, (Heinzen et al., 1996; Sauer et al., 2005; Beare et al., 2009). Also, the CRV contains active lysosomal hydrolases and cathepsin D indicating that the maturing vacuole fuses with lysosomal compartments (Voth and Heinzen, 2007). In addition, it has been demonstrated the participation of several SNAREs (Vamp3, Vamp7, and Vamp8, Syntaxin 17) in homotypic and heterotypic fusion events in order to consolidate the replicative vacuole (Campoy et al., 2011, 2013; McDonough et al., 2013). It has been additionally shown that the *Coxiella* housing compartment also interacts with the autophagic pathway (Gutierrez et al., 2005; Romano et al., 2007; Newton et al., 2014; Winchell et al., 2014; Kohler et al., 2016). Likewise, two other proteins involved in autophagy such as Rab24 and Beclin1 (Munafó and Colombo, 2002; Vázquez and Colombo, 2010) are also recruited to the CRV. Furthermore, induction of autophagy favors CRV development (Gutierrez et al., 2005; Romano et al., 2007).

Autophagy is a catabolic pathway that allows the degradation and recycling of intracellular components (organelles and proteins) in double membrane vesicles called autophagosomes (Shibutani and Yoshimori, 2014). It is believed that these structures sequester cytosolic material nonspecifically, but there is also a selective autophagic degradation process of various subcellular structures, including protein aggregates, mitochondria, as well as microbes (Tanida, 2011). A critical role for ubiquitin in this process has been demonstrated since adaptor proteins mediate the binding of autophagosome-associated ubiquitin-like proteins (i.e., LC3/GABARAP proteins) to ubiquitinated cargo, targeting the proteins to the autophagosome (Dupont et al., 2010). One of these ubiquitin binding proteins involved in selective autophagy is p62 (sequestosome 1; SQSTM1) which is an LC3-interacting partner and it is usually degraded by autophagy (Bjørkøy et al., 2005; Komatsu and Ichimura, 2010). Another important autophagy receptor is NDP52 (Nuclear Dot Protein 52 kDa) that shares with p62 the ability to bind LC3 and ubiquitinated cargo simultaneously (Perrin et al., 2004; Birmingham and Brumell, 2006). However, the specific roles of p62 and NDP52 are not fully understood. Recent reports have shown that reducing NDP52 expression resulted in a decrease in the number of autophagosomes containing *Salmonella enterica* (Thurston et al., 2012). It is important to mention that NDP52 is capable of directing the formation of an autophagic membrane around ubiquitinated bacteria in the cytosol to allow their clearance by the autophagy machinery (Thurston et al., 2009; Verlhac et al., 2015).

Galectins belong to a family of proteins highly conserved through evolution involved in several biological events. They are able to recognize specific carbohydrates in complex macromolecules located on cell membranes or in the extracellular matrix (ECM) through a carbohydrate recognition domain (CRD), which interacts with the structure (Gal β1 → 4 GlcNAc). According to the arrangement of their carbohydrate binding domains, galectins are classified into subfamilies: (a) Galectins “proto-type”: include galectins 1, 2, 5, 7, and 10. These proteins act as homodimers of two identical CRDs. (b) Galectins “chimera-type”: perform their function through a dual interaction. They are contacting carbohydrates through a CRD located at the carboxyl-terminal domain and also they interact with other ligands through their amino-terminal domain rich in proline, glycine and tyrosine. The only member of this family is Galectin-3 (Gal3). (c) Galectins with repetitive sequences (“tandem repeat type”): exhibit two structurally distinct CRD, giving them the capability of interacting with dissimilar carbohydrates. This group includes galectins 4, 6, 8, and 9.

Most galectins functions are performed extracellularly. These proteins are involved in processes of immunomodulation, cell adhesion, growth regulation, inflammation, embryogenesis, reproduction, metastasis, proliferation, and “splicing.” It has been observed that these carbohydrate binding proteins exert their biological effects through specific recognition-linked sugars in membrane receptors and extracellular glycoproteins (Rabinovich and Toscano, 2009; Vasta, 2013).

The mechanism of synthesis and release of galectins is not yet known. They accumulate in the cytoplasm before being secreted by a leader-peptide-independent pathway. Several studies have presented evidence that Gal3 accumulates in structures in close proximity to phagosomes containing *Shigella* or *Listeria* (Dupont et al., 2009; Paz et al., 2010). These bacteria lyse their phagosome, arguing that Gal3 may function as a danger receptor for membrane damages (Dupont and Lafont, 2009). It has been found that inflammasome components are associated with the *Shigella*-containing vacuole remnant membranes (marked with Gal3) that are driven to the autophagic machinery impairing the inflammatory response (Dupont et al., 2009). It was shown that a set of galectins, especially Galectin 8 (Gal8), recognize injured pathogen-containing vacuoles. Interestingly, at early infection times, Gal8 arbitrates the recruitment of NDP52 but at later times of infection ubiquitin is responsible for the recruitment of NDP52 (Thurston et al., 2009). Thus, this study highlights the recruitment of NDP52, mediated by two different molecules in a sequential fashion, to ensure the proper clearance of cytoplasmic *Salmonella*.

In the present study, we document, using several approaches, damages of the CRVs. Notably, we have found that several members of the galectin family interact with the *Coxiella burnetii*-containing vacuole, at different times of infection. Our results also indicate that adaptor molecules involved in autophagy-related signaling pathways associate to the limiting membrane of the CRV, likely to control distinct host cell responses upon pathogen infection. In addition, our results demonstrate for the first time that in a population of *Coxiella*-containing vacuoles the CRV membrane is damaged likely causing the leaking of protons and, as a consequence, a transient modification in its acidic pH. We show that impairing autophagy decrease the number of acidic bacteria-containing vacuoles. We propose that the autophagic pathway favors *Coxiella* infection by contributing to the repair of the transiently damaged replicative compartment.

## Results

### The CRVs present heterogeneous acidic characteristics

It has been previously demonstrated that *C. burnetii* transits the endocytic and autophagic pathways generating an acidic and replicative niche with phagolysosomal characteristics which develops in a large CRV at 48 h of infection (Akporiaye et al., 1983; Maurin et al., 1992; Heinzen et al., 1999; Grieshaber et al., 2002).

**Figure 1:**
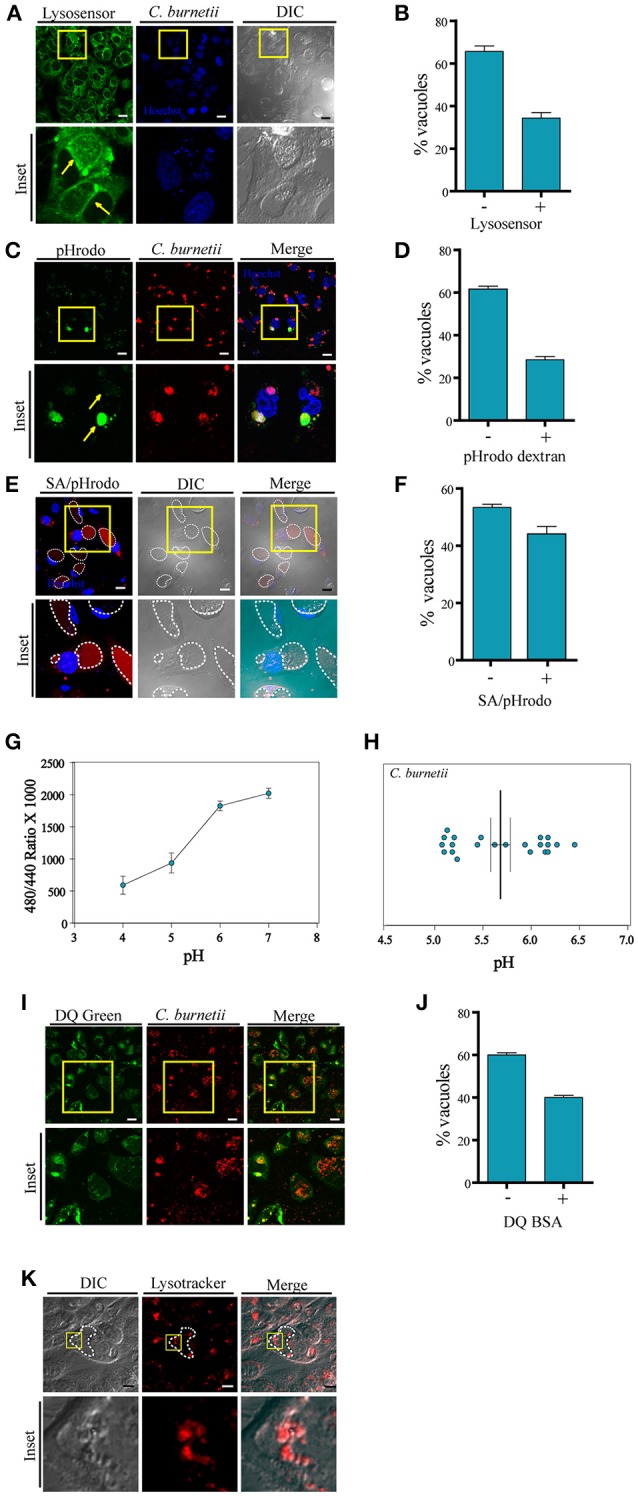
**Labeling with pHrodo indicates heterogeneousness of the pH of the CRVs. (A)** CHO cells were infected *with C. burnetii*. After 48 h, the cells were incubated with Lysosensor green, and analyzed by confocal videomicroscopy. **(B)** Quantification of the percentage of the vacuoles Lysosensor positive or negative. **(C)** CHO cells were infected with *C. burnetii*. After 48 h, the cells were incubated with pHrodo dextran (green), and analyzed by confocal videomicroscopy. Yellow arrows indicate the differences in fluorescence intensity in two different vacuoles. **(D)** Quantification of the percentage of the vacuoles pHrodo dextran positive or negative. **(E)** CHO cells were infected with *C. burnetii*. After 48 h, the cells were incubated with pHrodo coupled to dead *S. aureus* (SA/pHrodo) and analyzed by confocal videomicroscopy. **(F)** Quantification of the percentage of the vacuoles pHrodo positive or negative. **(G)** CHO cells were infected with *C. burnetii* for 48 h before loading for 20 h with Oregon green dextran. The standard curves were generated by measuring the ratio values for the lumenal space of *C. burnetii*'s vacuole as described for each of the six pH standards. These curves were used to determine the pH within both the Vero cell cytoplasm and the chlamydial inclusion. **(H)** Experimental ratio values were plotted against the standard curves and of *C. burnetii* replicative vacuoles were calculated. We determined the pH was 5.68 ± 0.3 (*n* = 22) for the *C. burnetii* replicative vacuole. **(I)** CHO cells were infected with *C. burnetii*. After 48 h, cells were incubated with DQ-BSA green and analyzed by confocal videomicroscopy. **(J)** Quantification of the percentage of the vacuoles DQ green positive or negative. The data represent the mean ± S.E.M. of at least three independent experiments (*n* > 100 cells/group). **(K)** CHO cells were infected with *C. burnetii*. After 48 h, the cells were incubated with LysoTracker red and analyzed by confocal videomicroscopy. Scale bar: 10 μm.

We and others have used LysoTracker to label the CRV indicating that these vacuoles have an acidic internal environment. To assess whether the entire population of CRVs posesses the same internal acidic characteristics, infected CHO cells were incubated with LysoSensor green. This membrane permeant dye increases its fluorescence in acidic environments. Living cells labeled with this compound were analyzed by confocal microscopy. To our surprise, we observed that several CRVs were indeed positive for LysoSensor but most of them displayed less or almost no fluorescence (Figures 1A,B), suggesting that the CRVs did not present homogeneous acidic properties at the time of observation.

Given these striking observations, a different experimental approach was used to assess the acidic pH inside the CRV. pHrodo is a compound that is almost non-fluorescent at neutral pH, and fluoresces brightly in an acidic environment. Thus, this pH marker allows detecting acidification or neutralization events by monitoring variations in its fluorescence intensity. Therefore, we first verified that the dextran/pHrodo beads that we were using actually responded to the changes in pH, as expected. As shown in the Figures S1A,B, the beads displayed a clear fluorescence intensity dependency according to the pH of the media. It is well-known that the *Coxiella* vacuole is highly fusogenic and readily fuses with other endosomes or phagosomes (e.g., bacteria-containing compartments). Therefore, we allowed the cells to endocytose pHrodo dextran or to phagocytose pHrodo-conjugated heat-killed *Staphylococcus aureus* (SA/pHrodo). CHO cells were infected with *C. burnetii* and incubated with pHrodo dextran or SA/pHrodo. The acidification of the CRV was visualized by fluorescence live cell imaging. As depicted in Figures 1C–F, pHrodo fluorescence displays heterogeneity between CRVs, suggesting different populations of these vacuoles depending on the pH. Our results indicate that a large percentage (more than 50%) of the vacuoles have a pH 7 or above because their low fluorescence intensity (Figures S1D–E). Interestingly, these differences were not dependent on the vacuole diameter (Figure S1C), suggesting that the fluorescence variability was not due to a dilution of the fluorochrome in the large CRV. Therefore, we can infer that there are different populations of CRVs with respect to their pH and that the possible transient loss of acidity in some of them seems to be independent on the size of the vacuole.

In order to assure that the large majority of the vacuoles has incorporated the pHrodo dye, we did some additional controls to the experiments described above. CHO cells were incubated with SA/pHrodo. The *S. aureus* particles were previously incubated with Hoechst to label the bacteria. Cells were analyzed by confocal live imaging (Figure S1D). The percentage of *S. aureus* positive vacuoles (labeled with Hoechst) as well as vacuoles pHrodo positive or negative was determined. As shown in Figure S1E, approximately 90% of the *C. burnetii vacuoles* contained *S. aureus* particles. However, only 40% of them were labeled with pHrodo, indicating that the pH of the rest of the vacuoles was not acidic. We also observed that the pHrodo fluorescence was dispersed throughout the CCV and not associated with the staph particle. We think this phenomenon is likely due to the release of the dye from the bacterial particles inside the internal environment of the CCV through a mechanism that remains to be determined.

On the other hand, CHO cells were infected with *C. burnetii* for 48 h. Then, the cells were co-incubated with Texas red dextran (red), as a control for dextran particles incorporation, and pHrodo dextran (green). Samples were analyzed by confocal microscopy (Figures S1F–G). The percentage of vacuoles Texas red dextran positive (red) or both Texas red dextran and pHrodo dextran positive (red and green) was determined. As indicated in Figure S1G whereas 100% of the *Coxiella* vacuoles have incorporated Texas red dextran, only 35–40% of the vacuoles were also labeled with pHrodo green. This result clearly indicates that the dextran particles were able to reach all the *Coxiella* vacuoles but only a proportion of those were labeled by pHrodo, indicating that a fraction of the vacuoles were not acidic.

To further verify the pH of the *C. burnetii* vacuole, we used ratiometric techniques. The *C. burnetii* vacuole was loaded with Oregon green dextran via fluid-phase endocytosis which has spectral properties that respond to low pH. *C. burnetii* infected CHO cells were loaded for 20 h with Oregon green dextran at a concentration of 1 mg/ml. The pH for the vacuole was calculated to be 5.68 ± 0.3 (*n* = 22; Figure 1G). We found that there are two different populations in *Coxiella* vacuoles pH, as represented in Figure 1H, supporting the existence of intact vacuoles with acidic pH around 5.2 and transient damaged ones with less acidic pH. This experiment was performed in CHO cells whereas in Akporiaye et al. (1983) and Maurin et al. (1992), macrophages were used. In Akporiaye et al. (1983), the experiment was performed with *C. burnetii* phase I in J774 cells and authors determined that the pH was 5.21 ± 0.07, whereas in Maurin et al. (1992), the pH in infected P388DI cells was 4.85 ± 0.3. On the other hand, in Vero cells, the pH of *C. burnetii*'s vacuole was 4.88 ± 0.14 as shown in the study by Grieshaber et al. (2002). Thus, it is possible that the differences observed in the measurement of the pH in the mentioned papers can be due to the cell type and/or the *Coxiella burnetii* type used.

To assess the degradative properties of the CRV, CHO cells were infected with *C. burnetii* for 48 h and then incubated with DQ-BSA green, a fluorescent marker for degradative compartments. Cells were visualized by fluorescence live cell imaging. As depicted in Figures 1I,J populations of CRVs labeled or not with DQ-BSA green were observed, in agreement with the existence of heterogeneous populations of the *Coxiella* replicative niche.

In addition, we performed a staining of infected *C. burnetii* CHO cells with LysoTracker (red). Consistently with the non-homogeneous distribution of the LysoTracker labeling, the presence of small vesicles inside the CRV marked by both probes was clearly observed (Figure 1K), confirming the multivesicular nature of the *Coxiella*-vacuole.

All together these results clearly indicate that at a given time point during infection, the CRVs are highly heterogeneous not only regarding to their acidic pH but also degradative properties.

### The CRV membrane is permeable to cytoplasmic macromolecular markers

We reasoned that the presence of different populations of *C. burnetii*'s vacuole with respect to their pH indicated that these vacuoles were damaged allowing passive movements of molecules across the CRV membrane. We hypothesized that the observed drop in acidity was a consequence of the leaking of protons through a damaged limiting CRV membrane.

**Figure 2:**
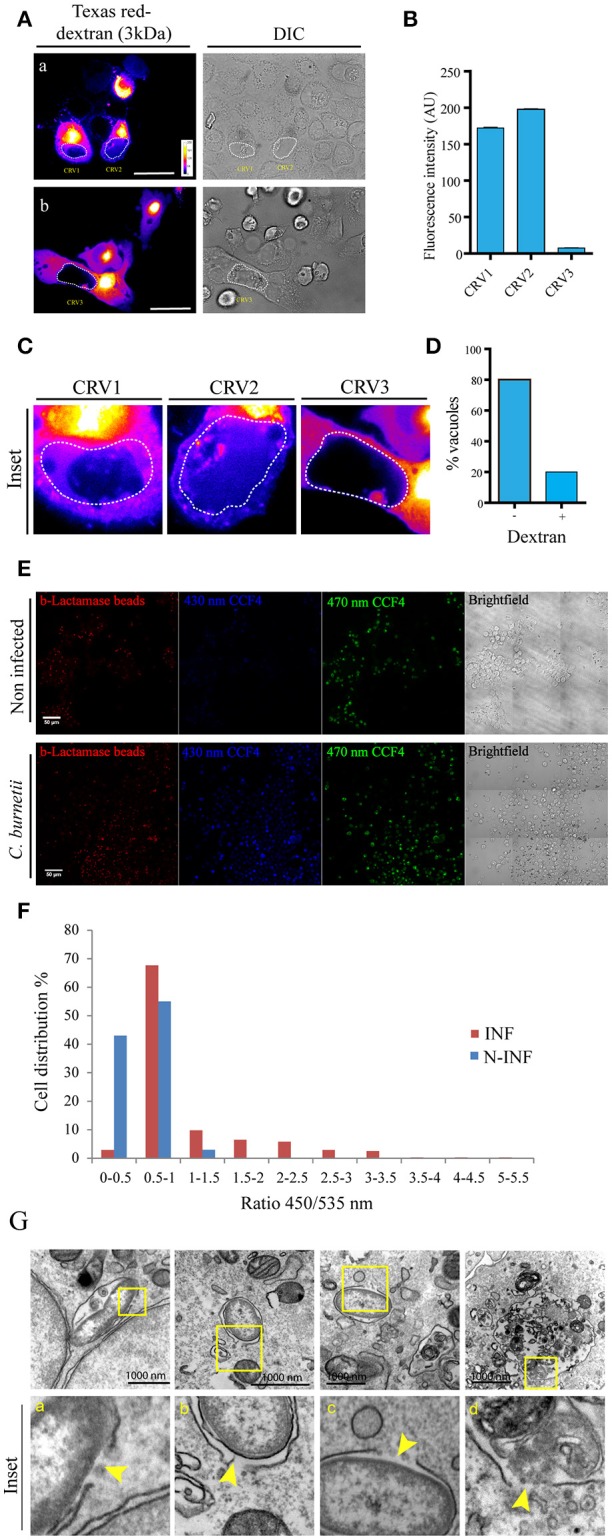
***Coxiella*** **damages the endo/phagosomal membranes in infected cells. (A)** CHO cells were infected with *C. burnetii* for 48 h and subsequently microinjected with 3 kDa Texas Red-tagged dextrans. Cells were immediately visualized by confocal videomicroscopy. **(B)** Quantification of the fluorescence intensity of the CRVs indicated in **(A)**. **(C)** Insets of the vacuoles CRV1, CRV2, and CRV3 shown in **(A)**. **(D)** Quantification of the percentage of vacuoles dextran positive or negative in microinjected cells. **(E)** CHO cells were infected with *C. burnetii* for 48 h and loaded with the CCF4 substrate for 2 h. Afterwards, cells were allowed to internalize 1 μm latex beads coupled with β-lactamase and imaged by fluorescence wide field confocal microscope. Picture acquisition was done randomly and automatically for each condition on 36 fields to follow the FRET signals and the fluorescent beads. Representative pictures were chosen with the following channels: β-lactamase beads (red), CCF4 cleaved probe 450 nm (blue) and CCF4 intact probe 535 nm (green). Scale bar: 50 μm. **(F)** Histogram representing the outcome obtained via specialized algorithms using the ImageJ software. The individual cells are distributed in function of their ratio of the intensities in the 450 and 535 nm channels. INF: *Coxiella* infected cells; N-INF: uninfected cells. The plots are representative of 3 independent experiments. **(G)**. CHO cells were infected with *C. burnetii* for 24 h and then processed for TEM. (a–d) show a bacterium inside a membrane disrupted phagosome. Arrowheads show the point where the membrane is damaged. The insets (a–d) show the higher magnification images of the structures shown in the corresponding top panel.

To ascertain whether *C. burnetii* containing vacuoles were permeable to cytoplasmic markers in a live cell system, soluble dextran of 3 kDa coupled to the Texas Red fluorochrome was microinjected into the cytoplasm of CHO cells previously infected with *C. burnetii* for 48 h. Vacuolar membrane permeability can be measured through the access of dextrans loaded into the cytoplasm to the CRV. The presence of dextran inside the vacuole was assessed by determining the fluorescence signal inside the CRV, using a spinning disk microscope. For each image of microinjected cells, a sequential optical series (z series) and time lapse images were recorded to determine the signal inside the CRV. Tagged dextrans of 3 kDa in size were able to gain access to vacuoles containing live *C. burnetii*. Figure 2A shows two images of one optical slice of the z serie and time lapse taken in each microinjection experiment. Figure 2A shows that Texas red dextran entered the CRVs labeled as CRV1 and CRV2. In contrast, the fluorescent marker was excluded from the CRV3 depicted in Figure 2B. The quantification of the fluorescence intensity inside each vacuole is depicted in Figure 2B. The differences among the three selected CRVs are clearly depicted in the insets shown in Figure 2C. The percentage of vacuoles accessed by the microinjected dextran is shown in Figure 2D. Thus, 20% of the vacuoles presents a relatively substantial damage of the limiting membrane. As a control of microinjection, a 200 kDa dextran was microinjected in the cytoplasm of infected CHO cells and the cells were visualized by confocal imaging as before. As depicted in the Figure S2A, this dextran could not get into de CRV, which means that vacuoles were not permeable to this size of molecules and that the microinjection procedure itself was not damaging the CRV.

In order to assure that the vacuoles are permeable, we had to adapt a FRET based reporter assay that was previously used to monitor the intracellular localization of *Salmonella* and *Shigella* species (Keller et al., 2013). In the host cell, membrane permeable CCF4-AM molecules diffuse freely across the cellular plasma membrane, gain access to the cytoplasm and are excluded from endosomes and other organelles by anion conversion into CCF4-AM upon cytosolic esterase action. The use of this probe relies on the bacterial expression and exposure of beta-lactamase. When the beta-lactamase gets in contact with the cytosolic substrate there is a switch in the FRET signal from 535 nm (green) to 450 nm (blue) upon 405 nm excitation, indicating cleavage of CCF4-AM. The change in the FRET signal indicates rupture of the phagosome/vacuole membrane. Ray et al. (2010). For our assay a modified approach was used. CHO cells were infected with *Coxiella* for 48 h and allowed to internalize 1 μm latex beads coupled with beta-lactamase. Afterwards, cells were incubated with the CCF4 substrate which is loaded in the cytoplasm. As previously demonstrated for *M. marinum* (Simeone et al., 2012) and other bacteria (Ray et al., 2010), when the beta-lactamase gets in contact with the cytosolic substrate there is a switch in the FRET signal from 535 nm (green) to 450 nm (blue), indicating rupture of the phagosome/vacuole membrane. As shown in the Figure 2E almost all the *Coxiella* infected cells (INF) turned blue whereas the uninfected (N-INF) cells remained green. The quantification of these results, using an algorithm segmenting individual cells and measuring both fluorescent channels, is shown in Figure 2F. High ratios (above 0.5) indicate the damage of the phagosome membrane as clearly depicted in the panel. As shown in the Figures S2B,C some of the incorporated beta-lactamase beads (red) can be visualized in a *Coxiella*-containing compartment (green).

In addition, we have also performed transmission electron microscopy of CHO cells infected with *C. burnetii* at early times of infection (24 h). In Figure 2G, we can observe several disruptions in both a *Coxiella*-containing phagosome membrane as indicated by yellow arrowheads.

Altogether these results noticeably indicate that the CRV surrounding membrane integrity is affected and permeable to cytoplasmic loaded compounds.

### Galectins are recruited to *C. burnetii*-containing compartments at different infection times

**Figure 3:**
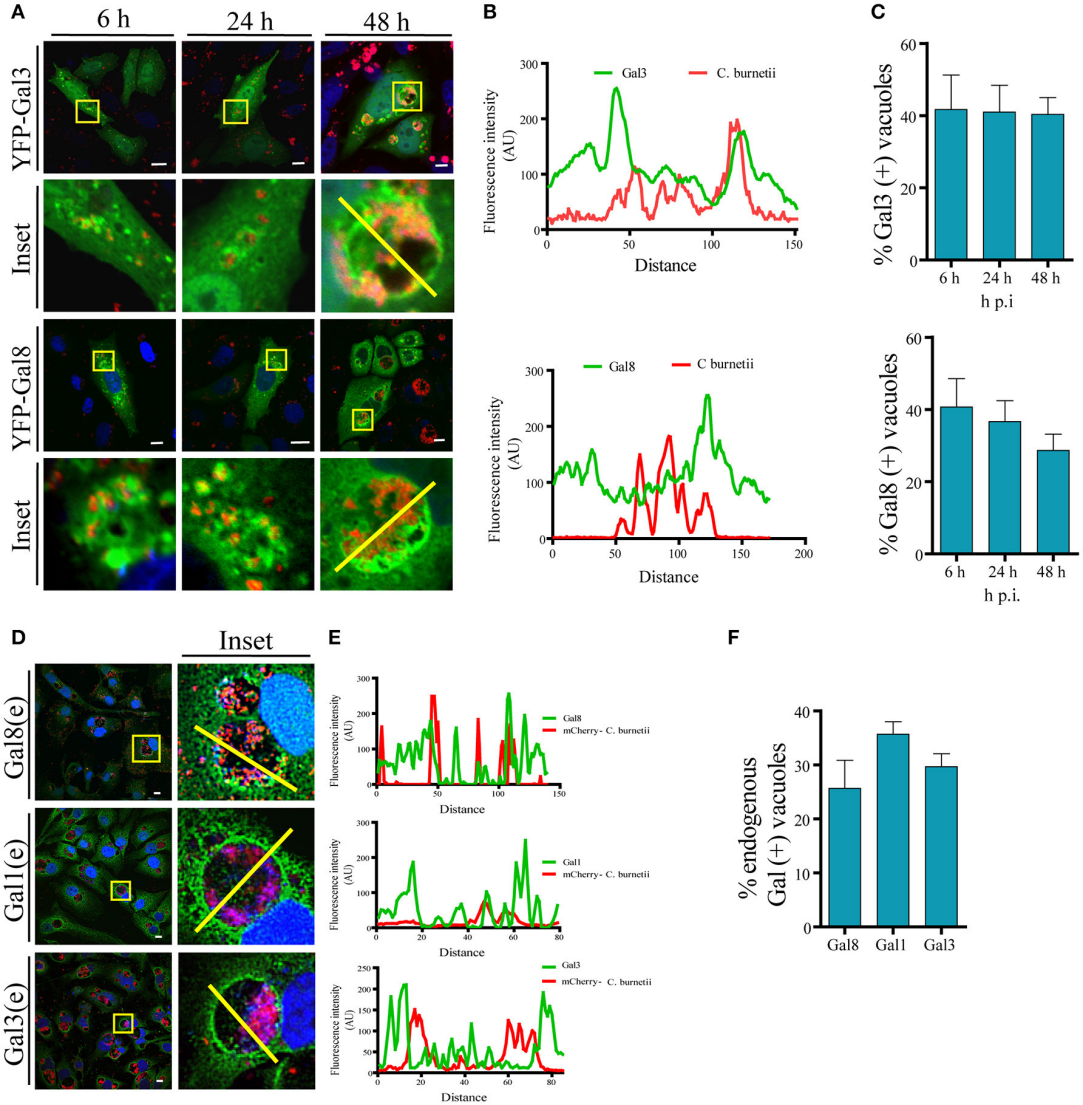
**Galectins decorate the CRV membrane. (A)** CHO cells were transiently transfected with YFP-Gal3 and -Gal8 and then infected with mCherry-*C. burnetii*. At 6, 2,4, and 48 h of infection, cells were fixed and analyzed by confocal microscopy. **(B)** Fluorescent intensity profiles along the yellow line depicted in the corresponding inset of **(A)** (YFP-Gal3 and -Gal8). **(C)** Quantification of the percentage of the CRVs labeled with YFP-Gal3 and -Gal8 from images like the ones depicted in **(A)**. The data represent the mean ± S.E.M. of at least three independent experiments (*n* > 50 cells/group). **(D)** CHO cells were infected with *C. burnetii* for 48 h, fixed and subjected to indirect immunofluorescence using antibodies against Gal1, Gal3, and Gal8 to detect the endogenous proteins (green), and an anti-*Coxiella* to detect the bacterium (red). Cells were analyzed by confocal microscopy. **(E)** Fluorescent intensity profiles along the yellow line depicted in the corresponding inset of **(D)**. **(F)** Quantification of the percentage of CRVs labeled with each of the galectins in images like the ones depicted in **(D)**. The data represent the mean ± S.E.M. of at least three independent experiments (*n* > 50 cells/group). Scale bar: 10 μm.

It has been reported that *C. burnetii* transits through the endocytic pathway, which delivers the bacteria to the low pH environment of a lysosome. It is known that CRV maturation to a phagolysosome takes approximately 2 h. Since galectins are considered useful molecules to detect damage of intracellular membranous compartments, we were interested in determining whether, at some time point during infection, the CRV membrane integrity was altered as suggested by the experiments described above. It has been reported that Gal3 and Gal8 detect membrane damage in bacteria's vacuoles like *Shigella* and *Salmonella* (Dupont et al., 2009; Paz et al., 2010; Thurston et al., 2012). Thus, we analyzed the association of these galectins with *Coxiella* phagosomes at early infection times (i.e., 6 h) and later infection times once the large vacuole is generated (i.e., 24, 48 h). To this aim, we overexpressed these galectins in CHO cells and infected cells with *C. burnetii*. At different times of infection (6, 24, and 48 h), cells were fixed and analyzed by confocal microscopy as shown in Figure 3A. Figure 3B shows the fluorescence intensity diagram along the yellow line across the CRV depicted in Figure 3A inset. We could observe galectins distributed at the limiting membrane and in the interior of the CRV as is shown in the Figure S3A, using Structured Illumination Microscopy (SIM) image of a transfected GFP-Gal3 CHO cell infected with *C. burnetii*. The quantification along the time of infection is depicted in Figure 3C, indicating that YFP-Gal8 and -Gal3 were clearly recruited to a population of the CRVs (30–40%) at all times of infection analyzed. We also screened a panel of other human galectins for their presence at the *C. burnetii*-containing vacuole and we determined whether the interaction of these galectins with the CRV was dependent on the time of infection. For this purpose, CHO cells were transfected for 24 h with YFP-Gal4, YFP-Gal10, YFP-Gal1 and YFP-Gal9 and cells were infected for different periods of time. Figure S3C shows confocal images of the kinetic analysis for the Gal1, Gal4, Gal9, and Gal10. The population of *C. burnetii* phagosomes that recruited the tested galectins during the course of infection was also quantified (Figure S3D). As depicted, the colocalization between *C. burnetii* and YFP-Gal1 increased at 24 h of infection and it remained constant at 48 h. In contrast, in the case of YFP-Gal9 and -Gal4 the proportion decreased at later infection times whereas no colocalization along infection was observed in the case of YFP-Gal10.

**Figure 4:**
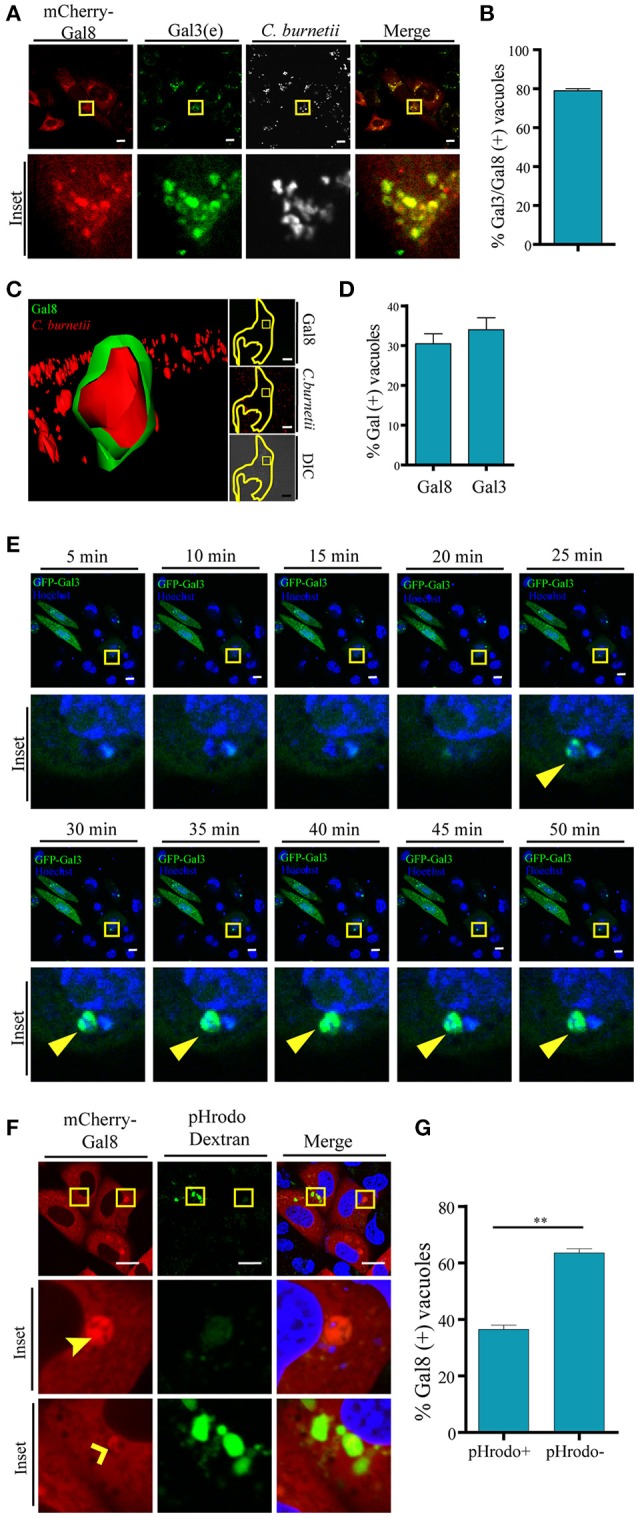
**Galectins are recruited to the *Coxiella* phagosomes at early times of infection. (A)** CHO cells were transiently transfected with mCherry-Gal8 and then infected with *C. burnetii* (white). At 6 h of infection, cells were fixed, and subjected to immunofluorescence against Gal3 [endogeneous (e), green]. The cells were analyzed by confocal microscopy. **(B)** Quantification of percentage of Gal3 positive vacuoles that colocalize with Gal8 in images like the ones depicted in **(A)**. The data represent the mean ± S.E.M. of at least three independent experiments (*n* > 50 cells/group). **(C)** CHO cells were transiently transfected with YFP-Gal8 and then infected with mCherry-*C. burnetii*. At 2 h of infection, cells were and analyzed by confocal videomicroscopy. The inset shows a multidimensional image constructed from a z-interval. **(D)** Quantification of the percentage of CRVs labeled galectins at 2 h of infection. The data represent the mean ± S.E.M. of at least three independent experiments (*n* > 50 cells/group). **(E)** CHO cells transiently overexpressing GFP-Gal3 were infected with *C. burnetii* (*MOI* = 50). The cells were analyzed by fluorescence microscopy for 2 h *in vivo*. Insets of the experiment show the sequences of the movie (every 5 min up to 50 min). Yellow arrows indicate the small *C. burnetii*-containing phagosome positive for GFP-Gal3. **(F)** CHO cells were transfected with mCherry-Gal8 and at 24 h post transfection were infected for 24 h with *C. burnetii*. *Coxiella*-infected cells were incubated with pHrodo dextran (green) for 3 h and visualized by confocal microscopy. **(G)** Quantification of the percentage of Gal8-positive vacuoles that were either pH rodo dextran positive (+) or negative (−). The data represent the mean ± S.E.M. of at least three independent experiments (*n* > 50 cells/group) (^**^P ≤ 0.01). Scale bar: 10 μm.

Since overexpressed Gal3, Gal8, and Gal1 interact with *C. burnetii* at different stages of vacuole development and persist along the infection process (48 h), we were interested in determining whether the endogenous proteins were also recruited at the CRV. Cells were infected with *C. burnetii* for 48 h and both bacteria and endogenous galectins were detected using specific antibodies. As shown in Figure 3D, we observed the accumulation of endogenous Gal3, Gal1, and Gal 8 at the CRV surrounding membrane (insets). The quantification of the percentage of CRVs positive for endogenous Gal3, Gal1, or Gal8 is depicted in Figure 3F. Our results clearly indicate that 25–30% of the CRVs are decorated by these galectins. Also, there is a large amount of colocalization between Gal3 and Gal8 at early times of infection (6 h; Figure 4A). CHO cells were transfected with mCherry-Gal8 and infected with *C. burnetii*. Then, the cells were fixed and subjected to immunofluorescence with specific antibodies against Gal3. The quantification of colocalization is depicted in Figure 4B, indicating that both galectins are recruited to the same CRV.

In addition, we were interested in determining the presence of galectins at earlier times of infection. For this purpose, CHO cells transfected with YFP-Gal8 or YFP-Gal3 were infected for 2 h with mCherry-*C. burnetii*. The cells were analyzed by fluorescence confocal videomicroscopy and a multidimensional image was constructed from a z-interval (Figure 4C). The image clearly shows the recruitment of YFP-Gal8 to a small phagosome containing just a bacterium, confirming that Gal8 associates to the *Coxiella* phagosome at very early times after infection. Figure 4D, shows the quantification of the recruitment of these galectins at 2 h of infection.

In order to dynamically image the recruitment of galectins to the *Coxiella* phagosome, CHO cells were transiently transfected with GFP-Gal3 and infected with *C. burnetii* incubated with Hoechst. The cells were analyzed by fluorescence confocal videomicroscopy. Figure 4E shows an infected cell with an early recruitment of Gal3 around a group of bacteria. The sequence shows images taken every 5 min in a sequence of the total time lapse analyzed from 0 to 50 min. Hence, there is a recruitment of Gal3 around bacteria starting at the time point 25 min of infection and remaining during the whole period of time analyzed while the phagosomes become larger.

Moreover, in order to assess whether some galectin-positive compartments have lost their acidity, CHO cells were transiently transfected with RFP-Gal8. Then, the cells were infected with *C. burnetii* for 24 h. Afterwards, the cells were incubated with pHrodo green dextran. The acidification of the CRV was visualized by fluorescence live cell imaging (Figure 4F). As depicted in the Figure 4G, the large majority of the mCherry Gal8-positive vacuoles are pHrodo negative. Nevertheless, *Coxiella*-containing vacuoles positive for Gal8 and pHrodo are also clearly observed. This can be interpreted as a transient membrane damage, hence neutralizing the vacuolar pH, before vacuoles recover acidification as membrane is repaired. In other words, at some point the vacuole was pierced and this damage was detected by galectins but since the damage is transient they recover the acidity but the CRV still remain positive for galectin. Thus, healing the membrane damage may contribute to the recovering of acidity necessary for the *Coxiella* metabolic activity.

In order to determine whether the galectin recruitment is specifically dependent on bacterial infection, a control experiment with an inert particle was performed. CHO cells were transfected with GFP-Gal3 and incubated with rhodamine beads for different times (2, 6, 24, 48 h), fixed and analyzed by confocal microscopy (Figure S4A). In addition, in a parallel experiment, endogenous Gal3 was determined in CHO cells incubated with FITC beads at the same time points (Figure S4C). As depicted in the graphs of fluorescence intensity along the yellow line across beads phagosomes, no recruitment of galectins to the beads-containing phagosomes was observed. Figures S4B,D show the quantification of the association of Gal3 to beads phagosomes.

### The recruitment of galectins is dependent on host cell glycans

**Figure 5:**
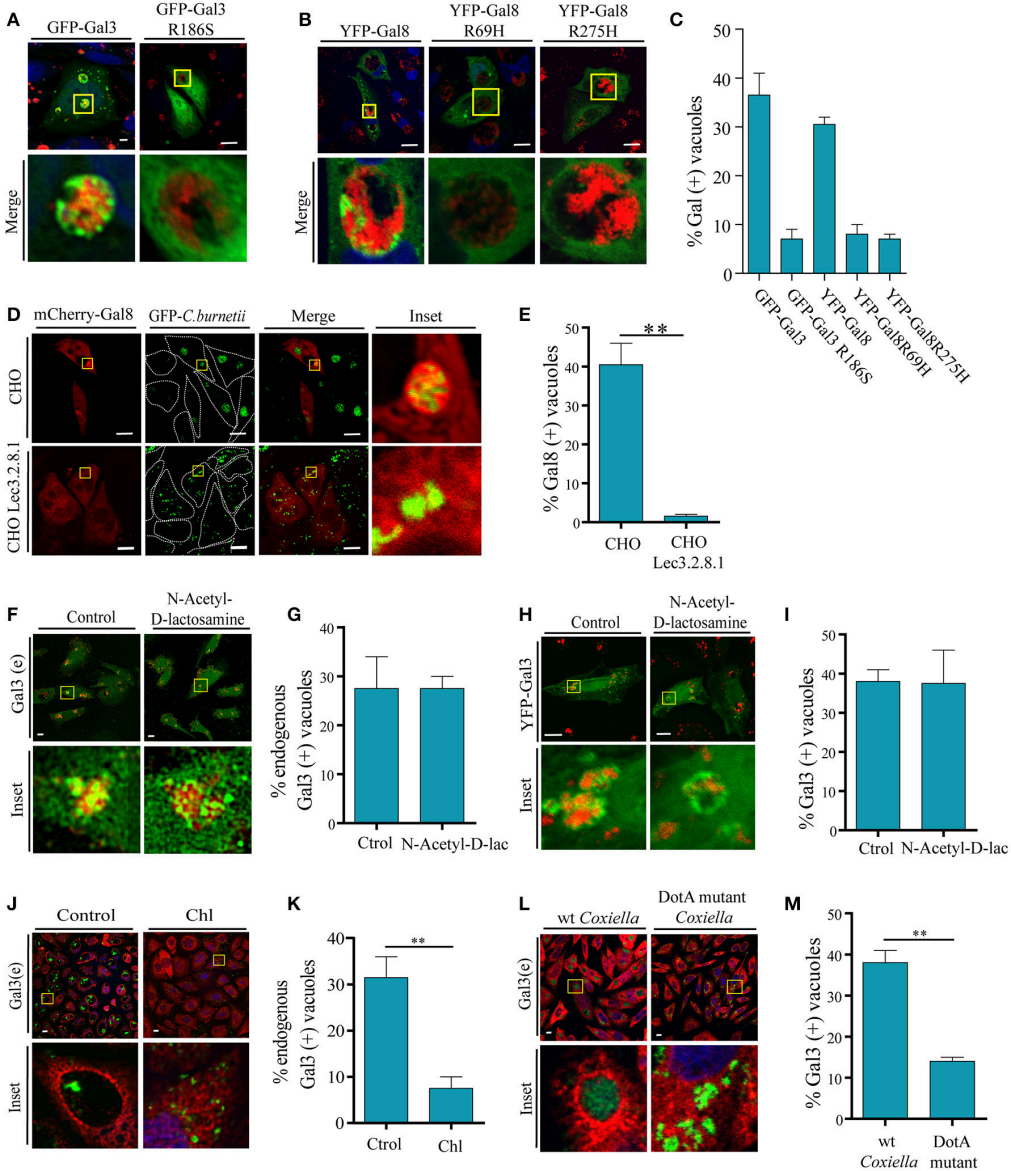
**Galectins recruitment is dependent on the binding to host glycans and on a bacterial secretion system. (A)** CHO cells were transiently transfected with YFP-Gal3 and YFP-Gal3 G186S and then infected with mCherry-*C. burnetii*. At 48 h of infection, cells were fixed and analyzed by confocal microscopy. **(B)** CHO cells were transiently transfected with YFP-Gal3, YFP-Gal8 R69H, and YFP-Gal8 R275H and then infected with *mCherry-C. burnetii*. At 48 h of infection, cells were fixed and analyzed by confocal microscopy. **(C)** Quantification of the percentage of CRVs labeled with each of the galectins in images like the ones depicted in **(A,B)**. The data represent the mean ± S.E.M. of at least three independent experiments (*n* > 50 cells/group). **(D)** CHO and CHO Lec3.2.8.1 cells were transiently transfected with mCherry-Gal8 and then infected with GFP-*C. burnetii*. At 48 h of infection, cells were fixed and analyzed by confocal microscopy. **(E)** Quantification of the percentage of CRVs labeled Gal8 from images depicted in **(D)**. **(F)** CHO cells were incubated with N-Acetyl-D-lactosamine. After 2 h, cells were infected with *C. burnetii* (red) maintaining the reagent in the culture medium and, at 48 h, the cells were fixed and subjected to immunofluorescence using specific antibodies against Gal3 (green) and analyzed by confocal microscopy. **(G)** Quantification of the percentage of CRVs labeled Gal3 in each condition (Control and incubated with N-Acetyl-D-lactosamine) from images like the ones depicted in **(F)**. **(H)** CHO cells were transiently transfected with YFP-Gal3 and then they were incubated with N-Acetyl-D-lactosamine. After 2 h, cells were infected and at 48 h, the cells were fixed and analyzed by confocal microscopy. **(I)** Quantification of the percentage of CRVs labeled Gal3 in each condition (control and incubated with N-Acetyl-D-lactosamine) from images like the ones depicted in **(H)**. **(J)** CHO cells were infected with either untreated (control) or treated *Coxiella* with 100 μg/ml of chloramphenicol (Chl) for 30 min, cells were incubated for 24 h, fixed and subjected to indirect immunofluorescence using specific antibodies against *C. burnetii* (green) and Gal3 (red). Cells were analyzed by confocal microscopy. **(K)** Quantification of the percentage of CRVs labeled with Gal3 from images like the ones depicted **in (J)**. **(L)** CHO cells were infected with GFP-*C. burnetii* and GFP-*C. burnetii* DotA mutant. At 48 h of infection, the cells were fixed and subjected to immunofluorescence using specific antibodies against Gal3 (red). The cells were analyzed by confocal microscopy. **(M)** Quantification of the percentage of CRVs labeled Gal3 from images like the ones depicted in **(G)**. The data represent the mean ± S.E.M. of at least two independent experiments (*n* > 50 cells/group) (^**^P ≤ 0.01). Scale bar: 10 μm.

The damage of the vacuole exposes host glycans which may cause Gal8 and Gal3 accumulation at the CRV. As indicated in the introduction, Gal3 has only one carbohydrate recognition domain (CRD) whereas Gal8 belongs to the group of “tandem repeat type” galectins that exhibit two structurally distinct CRDs, being able to interact with dissimilar carbohydrates. The requirement for carbohydrate binding by Gal8 and Gal3 was tested using point mutations in their respective CRDs. CHO cells were transfected with the corresponding carbohydrate-binding mutants YFP-Gal8 (R275H), YFP-Gal8 (R69H) and GFP-Gal3 (R186S). All these mutants carry a mutation in one critical aminoacid at the CRD so that they are not able to recognize carbohydrates. Afterwards, the cells were infected with *C. burnetii* and, at 48 h of infection they were fixed and analyzed by confocal microscopy (Figures 5A,B). As shown in the quantification depicted in Figure 5C, in contrast to the wt YFP-Gal8 or GFP-Gal3, none of the mutants did accumulate at the CRD, indicating that either the Gal3 CRD or both Gal8 CRD domains are absolutely required for recruitment to the *Coxiella* vacuoles.

Furthermore, to demonstrate that host glycans are indeed important for the recruitment of galectins to the CRV, we transfected CHO and CHO-Lec3.2.8.1 cells, which lack mature glycans, with mCherry-Gal8. Transfected cells were infected with GFP-*C. burnetii* and at 48 h of infection, cells were fixed and analyzed by confocal microscopy (Figure 5D). As shown in Figure 5E, recruitment of Gal8 to CRVs was severely impaired in the CHO-Lec3.2.8.1 cells. This indicates that the presence of mature glycans of the host cell is important for the recruitment of Gal8 to the CRV.

To assess whether Gal3 inside *C. burnetii*-containing vacuole may arrive from the plasma membrane when *C. burnetii* is phagocytosed, we utilized the reagent N-Acetyl-D-lactosamine, which blocks the interactions between Gal3 and its ligands at the cell surface. CHO cells were incubated with (N-Acetyl-D-lac) or without (Control) the inhibitor and after 2 h, the cells were infected with *C. burnetii* (red) for 48 h in the continuous presence of the inhibitor. Afterwards, the cells were fixed and endogenous Gal3 (green) was detected by immunofluorescence and analyzed by confocal microscopy (Figures 5F,G). The same experiment was performed with YFP-Gal3 transfected CHO cells (Figures 5H,I). We could observe the presence of Gal3 recruited to the vacuole in both control conditions and when incubated with the inhibitor (N-Acetyl-D-lac). This result indicates that the Gal3 present at the CRV does not come from the plasma membrane but from the cytoplasm.

### The recruitment of galectins is dependent on *C. burnetii* secretion system

It has been shown that *C. burnetii* possesses a type 4 secretion system (T4SS) termed Dot and Icm. Recent studies have demonstrated that Dot/Icm function is required for cytosolic delivery of several substrates which have been identified as proteins effectors important for intracellular replication, development of the large CRV and apoptosis protection of infected THP-1 cells. Nevertheless, it was found that translocation of Dot/Icm substrates is not required for axenic growth or uptake by host cells (Carey et al., 2011; Newton and Roy, 2011; Beare et al., 2012).

In previous reports, it has been demonstrated that T4SS function can be inhibited by chloramphenicol treatment. In order to assess whether the association between *C. burnetii* and galectins is an active phenomenon that depends on bacterial protein synthesis, *C. burnetii* was pretreated with 100 μg/ml of chloramphenicol for 1 h and CHO cells were then infected for 48 h, with the continuous presence of the antibiotic in the culture medium. Cells were fixed and subjected to a double immunofluorescence, using specific antibodies against *C. burnetii* and Gal3 (Figure 5J). Consistently with the results presented above, we observed in the control condition that a colocalization of about 30–40% of the CRV recruited this galectin, whereas with the chloramphenicol-treated bacteria the percentage of colocalization was significant smaller (around 10%; Figure 5K). This result clearly indicates that the recruitment of galectins depends on an active protein synthesis by *C. burnetii*. To actually demonstrate that galectin recruitment is dependent on T4SS, CHO cells were infected with GFP-wt *C. burnetii* or a DotA mutant GFP- *C. burnetii* (Tn292). This mutant carries an independent transposon insertion in the gene CBU_1648, which encodes DotA, an essential component of the *Coxiella* Dot/Icm secretion system. At 48 h of infection, cells were fixed and subjected to immunofluorescence against endogenous Gal3 (red). Similar to chloramphenicol treatment, cells infected with DotA mutant did not recruit Gal3 compared to control wt *Coxiella* (Figures 5L,M). These results show that *C. burnetii*'s Dot/Icm secretion system is important for the recruitment of galectins.

### NDP52 is present at the CRV and colocalizes with both LC3 and Gal3 or Gal8

It has been shown in previous publications that *Salmonella enterica* serotype *Typhimurium* and *Streptococcus pyogenes* are recognized by NDP52 and recruits polyubiquitinated proteins once in the cytosol (Thurston et al., 2009). NDP52 bridges LC3 and ubiquitin molecules around bacteria allowing the transport of cytosolic bacteria to the autophagic pathway. Based on these evidences, we next assessed the association of overexpressed RFP-NDP52 at the CRV. As shown in Figures S5A,B, the results indicate that the protein was found in 20–30% of the vacuoles generated after 48 h. In order to determine whether galectins also colocalized with NDP52 in the same *Coxiella* vacuole, YFP-Gal8 or YFP-Gal3 and RFP-NDP52 were coexpressed in CHO cells which were subsequently infected with *C. burnetii* for 48 h. As shown in Figures S5C,D, we found that around 80% of the galectin-positive CRVs accumulated NDP52. Furthermore, NDP52 also markedly colocalized with LC3 in the large vacuoles generated at 48 h infection (Figures S5E,F). The results suggest that galectin-dependent recruitment of NDP52 to the CRV is likely to participate to the further association of LC3.

The protein p62 (also known as SQSTM1) targets intracellular pathogens for degradation in autophagolysosomes. P62 is a receptor for cargo with multiple protein-protein interaction domains that binds ubiquitin and also LC3 (Komatsu and Ichimura, 2010). Because NDP52 was recruited to the *C. burnetii*'s CRV, we also evaluated the presence of p62 at the vacuole membrane. CHO cells were infected with mCherry-*C. burnetii* for 48 h and the cells were subjected to immunofluorescence with a specific antibody against p62. In the Figure S6A the association of p62 to the CRV membrane is depicted. Figure S6B shows the quantification of the percentage of vacuoles that recruit p62. Thus, we were next interested to also determine the presence of ubiquitin to the vacuoles. CHO cells were infected with *C. burnetii* for 48 h and then the cells were fixed. As depicted in Figure S6C, the vacuoles were decorated by ubiquitin (see quantification in Figure S6D) suggesting that *C. burnetii* recruits in an ubiquitin-dependent manner adaptors of the autophagy machinery.

### The population of CRV galectin positive vacuoles increases in cells with a defective autophagy pathway

In several previous publications it has been shown the association of LC3 to the large CRV (Berón et al., 2002; Gutierrez et al., 2005; Romano et al., 2007). Therefore, we next examined whether both Gal3 or Gal8 and LC3 were recruited to the same vacuole. To examine in details the respective distribution of galectins and LC3 during *C. burnetii* infection, cells were co-transfected with YFP-Gal3 or YFP-Gal8 and RFP-LC3 and they were infected with *C. burnetii* for 48 h. Figure S5G clearly shows the presence of either YFP-Gal3 or YFP-Gal8 and RFP-LC3 decorating the large CRV. The image shows the accumulation of LC3 (red) and galectins (green) at the surrounding CRV membrane but also in small structures inside the CRV that may represent small internal vesicles (Figure S5G insets). Figure S5H shows the quantification of LC3 positive vacuoles (from a total around of 30%) that colocalize with Gal8 or Gal3. We can conclude that in 48 h vacuoles, galectins and LC3 are both present suggesting that similar to other pathogens galectins may mediate the recruitment of LC3 to the CRV.

In order to assess whether the recruitment of Gal3 was modulated by the autophagic pathway, CHO cells overexpressing Gal3 were infected with *C. burnetii* duting 48 h. Then, the cells were subsequently incubated in full nutrient media (Control) or under starvation conditions (Stv) or with Rapamycin (Rap) to induce autophagy. As shown in Figure S5J, no major differences were observed in the recruitment of Gal3 in all the conditions tested.

**Figure 6:**
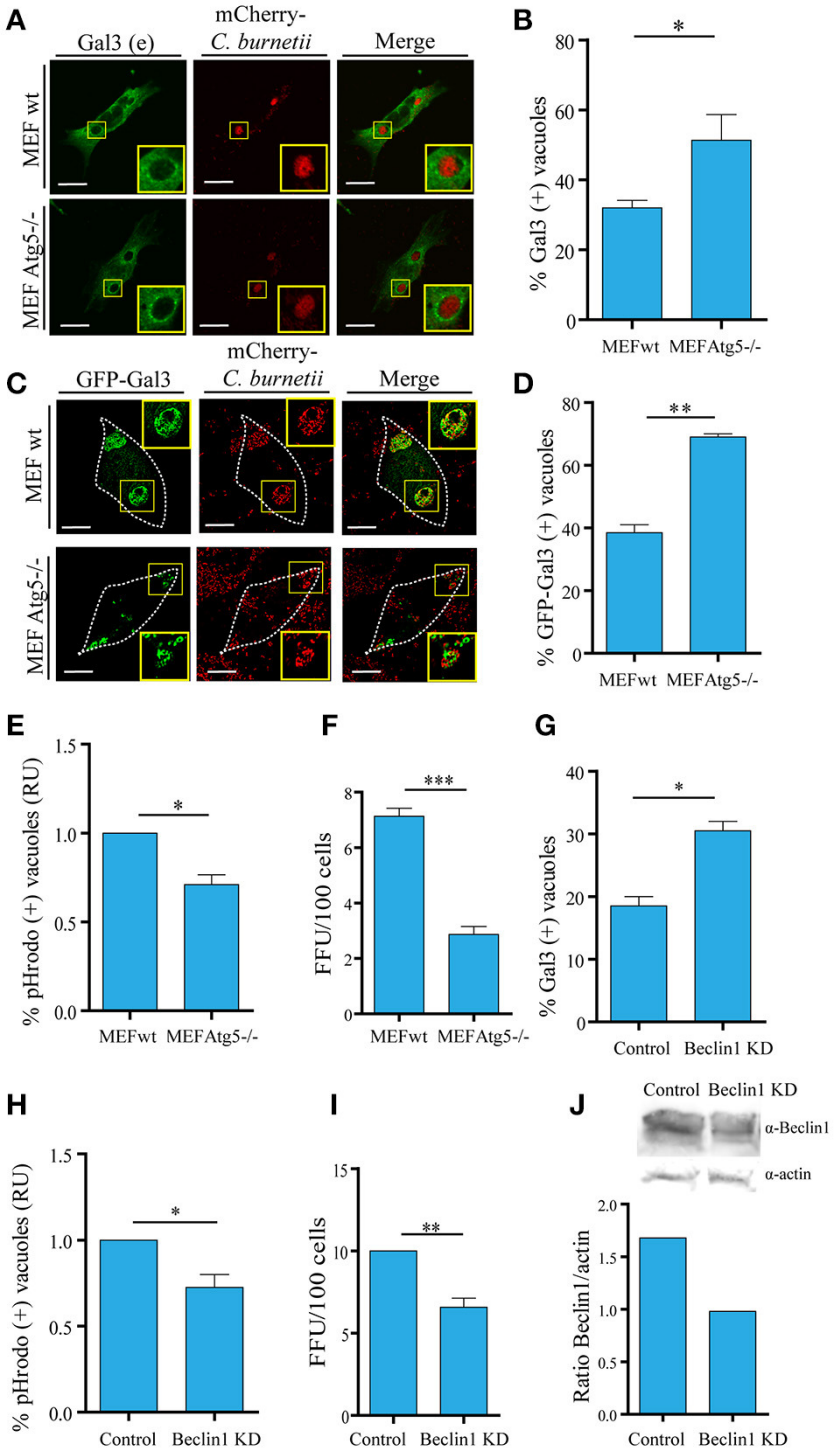
**Autophagy contributes to keep the integrity of the vacuolar membrane. (A)** WT and Atg5 −/− MEF cells were infected with *C. burnetii* (red) and, at 48 h of infection, cells were subjected to indirect immunofluorescence with specific antibodies against Gal3. **(B)** Quantification of Gal3-positive vacuoles in MEF wt and Atg5−/− cells is shown. **(C)** WT and Atg 5−/− MEF cells were transfected with GFP-Gal3. After 24 h post-transfection, cells were infected with *C. burnetii* (red) for 48 h. Cells were fixed, and examined by confocal microscopy. **(D)**. Quantification of Gal3-positive vacuoles in MEF wt and Atg5−/− cells is shown. Scale bar: 10 μm. **(E)** Quantification of the pHrodo-positive vacuoles in MEF wt and Atg5−/− cells infected with *C. burnetii*. After 48 h, cells were incubated with pHrodo, and analyzed by confocal videomicroscopy. Data represent the mean ± SEM of at least three independent experiments in which no <200 vacuoles were scored in each experiment (^*^P ≤ 0.05). **(F)** MEF wt and MEF Atg5−/− cells were infected with *C. burnetii* and following 48 h of infection to allow the development of the *Coxiella* vacuole. The cells were then lysed by sonication and the supernatant was diluted (1:100) and used to infect Vero cells. After 72 h of incubation (chase), cells were fixed and examined by fluorescence microscopy. Data represent the mean ± SEM of at least three independent experiments where at least 1,000 cells were scored in each experiment (^***^P ≤ 0.001). **(G)** HeLa cells were transfected with Beclin 1 KD plasmid using a double-hit protocol. Control and Beclin 1 KD cells were infected with *C. burnetii* and, at 48 h of infection, cells were subjected to indirect immunofluorescence with specific antibodies against Gal3. Cells were fixed, and examined by confocal microscopy. Quantification of Gal3-positive vacuoles in control and Beclin1 KD cells is shown. **(H)** Quantification of the pHrodo-positive vacuoles in HeLa control and Beclin1 KD cells infected with *C. burnetii*. Cells were treated and infected as described in **(G)**. After 48 h, cells were incubated with pHrodo and analyzed by confocal videomicroscopy. Data represent the mean ± SEM of at least three independent experiments in which no <100 vacuoles were scored in each experiment (^*^P ≤ 0.05). **(I)** HeLa cells were transfected with pEGFP or co-transfected with pEGFP and Beclin 1-KD plasmid using a double-hit protocol (see Experimental Procedures for details). After 24 h, cells were infected with *C. burnetii* and cultured for an additional 48 h period to allow the development of the large *Coxiella* vacuole. The cells were then lysed by sonication and the supernatant was diluted (1:100) and used to infect Vero cells. After 72 h of incubation (chase), cells were fixed and examined by fluorescence microscopy. Data represent the mean + SEM of at least three independent experiments where at least 1,000 cells were scored in each experiment (^**^P ≤ 0.01). **(J)**. (Top) Western blot of the assay described in **(G)** and (bottom) quantification of intensity of Beclin1 bands relative to actin.

To directly assess the role of autophagy in galectins recruitment, we used a mouse embryonic fibroblast (MEF) cell line harboring a knock-out for the *atg5* locus (*atg5* −/−). These cells lacking Atg5 are deficient in macroautophagy because this protein is essential for the early steps of autophagosome formation. To demonstrate the significance of the membrane damaged caused by *Coxiella* and the role of autophagy in this process, MEF wt and Atg5−/− cells were infected with *C. burnetii* and following 48 h of infection, the cells were fixed, subjected to immunofluorescence against Gal3 (red) and analyzed by confocal microscopy. As expected, in control cells, endogenous Gal3 was recruited to a population of the CRVs. However, in MEF Atg5−/− cells, a higher percentage of vacuoles decorated by endogenous Gal3 were observed, indicating that a larger number of CRVs were damaged and that autophagy is likely required for membrane repair (Figures 6A,B). Similar results were also observed when cells MEF wt or Atg5−/− overexpressing YFP-Gal3 were infected with *C. burnetii* confirming that this galectin was recruited to the vacuole in an autophagy-independent manner (Figures 6C,D). However, the number of CRV positive for galectin was dependent on a functional autophagy pathway.

As indicated above pHrodo is a fluorogenic dye that becomes highly fluorescent as the pH decreases, therefore, it is a useful probe to assess acidic compartments. To corroborate that in autophagy incompetent cells more vacuoles were actually damaged and, as a consequence, the acidity of the CRV was lost due to the leaking of protons, we used pHrodo as a marker of acidic vacuoles. For this purpose, MEF wt and Atg5−/− infected cells were incubated with pHrodo-conjugated to heat-killed *Staphylococcus aureus* to be internalized and visualized by fluorescence live cell imaging. As depicted in Figure 6E, in Atg5−/− cells, there was a decrease in the number of vacuoles labeled with the fluorogenic compound, suggesting that there were less acidic vacuoles in autophagy-deficient cells. This result supports the participation of autophagy in keeping the integrity of the *Coxiella* vacuole limiting membrane.

We next determined the importance of a functional autophagy pathway in *Coxiella* growth. To assess the role of autophagy in bacteria replication, a focus-forming unit (FFU) assay was performed as follows: MEF wt and Atg5−/− cells were infected with *C. burnetii* and after 24 h of infection, cells were scraped and lysed by sonication. Sonicates were diluted in infection medium (i.e., DMEM) and a monolayer of Vero cells were infected with a dilution of the obtained supernatant (1:100). After 72 h of incubation (chase), cells were fixed and examined by fluorescence microscopy (please see *Experimental Procedures* for more details). The FFU assay showed a marked decrease in the replicative capacity of *C. burnetii* in MEF Atg5−/− in comparison with wt cells, indicating an active role of autophagy in *Coxiella* growth (Figure 6F).

We have previously demonstrated that the autophagic protein Beclin 1 was recruited to the *Coxiella* replicative vacuole and that overexpression of this protein favors the development of the CRV (Vázquez and Colombo, 2010). In order to confirm the significance of the membrane damaged caused by *Coxiella* and the role of autophagy in this process based in the participation of Beclin1, we performed the silencing of this protein using a specific siRNA plasmid (Vázquez and Colombo, 2010). Control and Beclin 1-KD cells were infected with *C. burnetii* and following 48 h of infection, the cells were fixed, subjected to immunofluorescence against Gal3 and analyzed by confocal microscopy. As expected, in control cells, endogenous Gal3 was recruited to a population (20%) of the CRVs. However, in Beclin 1-KD cells, a much higher percentage of vacuoles decorated by endogenous Gal3 were observed (Figure 6G).

In order to determine the acidity of the vacuoles, control, and Beclin 1-KD infected cells were incubated with pHrodo green dextran and visualized by fluorescence live cell imaging. As depicted in Figure 6H, in Beclin1 KD cells, there is a decrease in the number of vacuoles labeled with the fluorogenic compound. To verify whether Beclin 1 was also involved in the replicative capacity of *Coxiella*, HeLa cells were transfected with a pEGFP plasmid as a control or co-transfected with a plasmid encoding a small interference RNA against Beclin 1 (pSuper Beclin 1-KD), in order to knockdown the endogenous Beclin in transfected cells (Figure 6J). After 24 h cells were infected with *C. burnetii* for additional 24 h. The replication capacity of the bacteria was determined by the FFU assay in cells subjected to Beclin 1 knockdown. As shown in Figure 6I, left panel, a significant decrease in bacteria replication was observed when Beclin 1 was silenced which indicates that this autophagic protein is involved in the generation of an appropriate environment for bacteria growth. Taken together, these results suggest that autophagy has an important role in keeping the integrity of the membrane vacuole favoring also bacteria replication.

## Discussion

Galectins are molecules that bind β-galactosides at the cell surface and are also present in the cytosol, which normally lacks complex carbohydrates. Therefore, galectins may be considered as danger signal (Dupont and Lafont, 2009) and/or pattern-recognition receptors that identify pierced or broken membranes. In the present work, we report the association of galectins to the CRV, at different times of infection. All together results presented in this study support that the CRV membrane is damaged and becomes permeable to molecules like galectins that recognize glycans present in the luminal side of the vacuolar membrane. Interestingly, different members of the galectin family were recruited in a differential extent according to the infection times. We have observed that Gal3, Gal9 and Gal8 are present at the vacuole membrane in a high percentage of CRVs from early times of infection. In contrast, Gal1 is mainly recruited at later times of infection. This suggests that there are differences in the composition of the carbohydrates present at the inner face of the *C. burnetii* vacuole which are specifically recognized by each galectin. The presence of Gal1 at later infection times is likely due to the more mature (i.e., lysosomal) characteristics of the CRV at 48 h. Our results are consistent with a previous publication by Randow and collaborators (Thurston et al., 2012), showing that damage of endosomes by *Salmonella* induces the formation of Gal3, Gal8 and Gal9 puncta, without presence of Gal1. In contrast, specific damage of lysosomes results in the recruitment of Gal1. Interestingly, our results indicate that Gal10 is not present at the *Coxiella* vacuole membrane during the whole infection period analyzed. It is known that Gal10 recognizes mannose ligands but does not recognize other type of carbohydrates such as N-acetyl-lactosamine molecules. Thus, the lack of Gal10 recruitment at the CRV is likely due to the absence of mannose residues at the *Coxiella* vacuole.

As mentioned above galectins are recruited to damaged membranes by their interactions with N-glycans that are exposed to the cytoplasm when the membrane of a vesicle is pierced. However, other intracellular functions have also been described for Gal3. It has been shown that Gal3 associates with Bcl-2 and is thought to participate in the inhibition of cellular death (Yang et al., 1996). Thus, although our present results point to Gal3 as a sensor of damage at the *Coxiella* limiting membrane, we cannot discard the possibility that Gal3 is exerting other functions at the *Coxiella* vacuole. Indeed, we have previously demonstrated that Bcl-2 is also recruited to the CRV and exerts a critical role in avoiding the death of the infected cells (Vázquez and Colombo, 2010). Future analyses would be necessary to actually determine other possible functions of Gal3 in *Coxiella* infection.

In this work, we focused on the analysis of two galectins, Gal3 and Gal8. These galectins are the best studied related to pathogens and damage of vacuole membranes, as shown in the case of *Shigella* (Dupont et al., 2009), *Salmonella* (Thurston et al., 2012), *Listeria* and a virus like Adenovirus (Maier et al., 2012). A pioneer work, identified the cytosolic protein Gal3 as a marker of membrane damage of phagosomes (Dupont et al., 2009; Paz et al., 2010) since the protein was recruited to membrane remnants of *Shigella*-containing compartments. In the case of *Coxiella*, we have observed that Gal3 is associated to the vacuole at the different times of infection analyzed (6, 24, 48 h). We have also demonstrated that the recruitment of galectins depends on the presence of mature glycans from the host cell and that the recognition of these glycans by galectins depends on intact CRDs. It is important to mention that our results also indicate that recruited galectins comes from the cytosol and not from the plasma membrane during the process of *Coxiella* internalization.

There is evidence that autophagy may function as a selective pathway that degrades protein aggregates and organelles. It has been shown that ubiquitination in conjunction with adapter proteins such as p62/SQSTM1, which allows the interaction between LC3 and ubiquitin, participates in the targeting to autophagy. We have shown the presence of host p62 and ubiquitin on CRV membranes suggesting that *C. burnetii* interacts with the host ubiquitination-autophagy interface during intracellular growth. Our results about the association of p62 to the CRV membrane are in agreement with recent findings showing the recruitment of p62 to the *Coxiella* parasitophorous vacuole (Winchell et al., 2014). Interestingly, this recruitment and the LC3 association, were dependent on a functional type IV secretion system (Winchell et al., 2014), which is also consistent with our observations that the recruitment of LC3 (Romano et al., 2007) and galectins (this report), is abolished by chloramphenicol treatment which hampers bacterial protein synthesis. We have also demonstrated here that a type IV secretion mutant was unable to recruit galectins to the *Coxiella* vacuole.

Our present results are also consistent with earlier report showing that the membrane remnants released after the rupture of *Shigella*-containing vacuolar membranes undergo protein polyubiquitination and subsequently are targeted to autophagy (Dupont et al., 2010). The role of p62 and/or NDP52 in selective autophagy of *Salmonella enterica* serovar *Typhimurium* (*S. typhimurium*) has recently been characterized (Zheng et al., 2009; Mostowy et al., 2011). It has been proposed that p62 and NDP52 act independently to drive efficient bacterial autophagy of *S. typhimurium* within *Salmonella*-containing vacuoles (Verlhac et al., 2015). In this work, we have shown that a population of *Coxiella* vacuoles is marked by NDP52, with a pattern very similar to ubiquitin. It remains to be determined if similar to *Salmonella* both adaptors participate independently or not to mediate the interaction with the autophagy pathway.

It is evident that once the CRV membrane is damaged or pierced, the vacuole would lose the protons and the acidic characteristics. To assess vacuole acidification, we have utilized LysoSensor and pHrodo, a novel fluorogenic dye that dramatically increases in fluorescence as the pH becomes more acidic. Our experiments using pHrodo directly demonstrate that different populations of CRVs exist. Indeed, some of them displayed high fluorescence intensity but some were not stained. These differences were not dependent on vacuole diameter and were not dependent on the presence or the absence of autophagy markers (i.e., LC3, not shown). Thus, our results suggest that some vacuoles have lost their protons and, as a consequence, they are not labeled by pHrodo. The permeability of the *Coxiella* membrane vacuole was demonstrated by the microinjection of small molecules such as 3 kDa dextran, in the cytoplasm of cells infected with *C. burnetii*. Our results indicate that a fraction of the *Coxiella* vacuoles were permeable to the cytoplasmic microinjected dextran as demonstrated by the accumulation of the fluorescent compound inside the vacuole. It is interesting to mention that in a publication by David Russell and collaborators, the authors found that dextran particles previously loaded into cells, were transferred from the cytoplasm to the *Leishmania mexicana* parasitophorous vacuole likely via an autophagy-dependent mechanism. However, this process required prolonged incubation times (i.e., 10 h) since at 4 h post-loading the dextrans remained mostly cytosolic (Schaible et al., 1999). Also, in a publication of Heinzen and Hackstadt (1997), Vero cells with large PVs harboring *C. burnetii* were microinjected with probes as small as 623 Da showing the CRV is impermeable to them. In contrast, in our system, the transfer of fluorescent dextran to the *Coxiella* vacuole was almost immediately after the microinjection into the cytoplasm which is more compatible with a damage of the *Coxiella* membrane. In addition, we have demonstrated by using a modified FRET assay, based in the detection of β-lactamase activity when the cytoplasm gets in contact with internalized beads coated with this enzyme, that the integrity of the CRV is altered in *Coxiella* infected cells.

It has been shown that lysosomes are also marked with Gal3 when they are damaged and, as a consequence, can be targeted to autophagy (Maejima et al., 2013). The induction of autophagy by damaged lysosomal compartments occurs after the loss of acidic content and release of lysosomal cathepsin D. This last evidence points to a role of autophagy in the sequestration of damaged lysosomes in order to allow the isolation of harmful material that may induce inflammatory responses. Indeed, it has been observed membranes around the lysosomes, suggesting that canonical autophagy eliminates damaged lysosomes. Once the rupture of the lysosome happens, the autophagosomes would selectively hijack damaged lysosomes. Ubiquitin is likely involved in the recognition of these damaged lysosomes. It is also feasible that autophagosomes fuse with lysosomes and restore the damaged compartments by repairing the holes in the membrane, resulting in the recovery of the acidic pH. This mechanism was also demonstrated in a Salmonella infection system. In a recent publication of Kreibich et al, it has been shown that autophagy participates in the repair of SCV membrane damage (Kreibich et al., 2015). This autophagy-dependent SCV sealing requires recruitment of factors such as optineurin and galectin 3 and the autophagy initiator factors ULK1, Beclin1, and ATG9, suggesting that LC3 is recruited to SCV by a canonical pathway (Kreibich et al., 2015).

Thus, we propose that a similar mechanism may take place in *Coxiella* infected cells, allowing the vacuole to restore its acidic properties and degradative capacity. Indeed, we propose herein that *C. burnetii*'s vacuole might restore its acidic environment by resealing the membrane. We have presented evidence that in MEF cells KO for Atg5, a critical protein involved in autophagy, a larger number of Gal3-labeled CRVs are detected, suggesting an increase in the number of damaged *Coxiella* vacuoles. In agreement with this, we have also found, based on the use of the pHrodo marker, a decrease in the number of *Coxiella*-containing acidic vacuoles. Therefore, our present results support a role for autophagy in membrane repair contributing to the integrity of the *Coxiella* replicative vacuoles.

In summary, in the present report we have uncovered an unexpected outcome of the *Coxiella* infection which is the damage of the *Coxiella*-containing vacuole causing that several CRVs do not maintain its acidic pH. We propose that autophagy may contribute to the resealing of the damaged membrane likely via fusion with autophagosomes and autolysosomes. Further studies are necessary to elucidate the molecular machinery involved in the damage and in these fusion events leading to the repair of the injured CRV membrane. Our study paves the way to investigate whether this phenomenon is also involved in other infectious process and beyond the infection field how general is the role of autophagy in membrane repair mechanisms.

## Experimental procedures

### Materials

Minimum essential Medium Alpha Medium (alfa-MEM) and Dulbecco's Modified Eagle Medium (D-MEM) were obtained from Gibco Laboratories (Invitrogen, Argentina); fetal bovine serum (FBS) was obtained from GIBCO BRL/Life Technologies (Buenos Aires, Argentina). Rabbit polyclonal anti-*Coxiella* antibody and mCherry-*Coxiella burnetii* were generously provided by Dr. Robert Heinzen (Rocky Mountain Laboratories, NIAID, NIH, Hamilton, MT, USA). Plasmids encoding YFP-Gal4, YFP-Gal9, YFP-Gal3, YFP-Gal10, YFP-Gal1, YFP-Gal8, and RFP-NDP52 were kindly provided by Dr. Felix Randow (MRC Laboratory of Molecular Biology, Hills Road, Cambridge, CB2 0QH, U.K.). Rabbit and mouse polyclonal antibodies anti-Gal3, -Gal1, -Gal8 and a plasmid encoding Gal3 were generously provided by Dr. Gabriel Rabinovich (Instituto de Biología y Medicina Experimental, INYME-CONICET, Buenos Aires, Argentina). The Beclin 1 KD plasmid was a generous gift of Dr. William Maltese (Medical University of Ohio, Toledo, Ohio, USA). Tandem fluorescent-tagged Galectin-3, tfGal3 was kindly provided by Dr. Tamotsu Yoshimori (Laboratory of Intracellular Membrane Dynamics, Graduate School of Frontier Biosciences, Osaka University, Osaka, Japan). mCherry-Gal8 was kindly provided by Dr. Mauricio Terebiznik (Department of Biological Sciences, University of Toronto, Toronto, Canada). *Coxiella*-GFP (Tn1832) and DotA mutant GFP (Tn292) were kindly provided by Dr. Matteo Bonazzi (Cell Biology of Bacterial Infections, UMR 5236 CPBS, Montpellier, France). CHO-Lec3.2.8.1 cells were kindly provided by Dr Pamela Stanley (Albert Einstein College of Medicine, New York, USA). Ubiquitin antibody was from Abcam.

### Fluorophore

Texas Red-tagged dextran (3 kDa), pHrodo™ Succinimidyl Ester, pHrodo™ Green dextran, 10,000 MW, LysoTracker, LysoSensor green DND-189 and DQ-BSA were purchased from Molecular Probes.

### Cell culture

Vero (ATCC, CCL-81), MEF (ATCC, SCRC-1040), and Chinese hamster ovary cells (CHO; ATCC, CCL-61) were grown on coverslips in D-MEM or α-MEM supplemented with 15% FBS, at 37°C in an atmosphere of 95% air and 5% CO2, in 24-well-plates to 80% confluence.

### Cell transfection

MEF and CHO cells were transfected with the plasmids (1 μg/μl) using LipofectAMINE 2000 reagent (Invitrogen, Argentina) as previously described in Campoy et al. (2013). Transfected cells were incubated for 24 h in DMEM and were infected with *C. burnetii* as described above.

### Propagation of *Coxiella burnetii* phase II cells

Obtaining *C. burnetii* was performed as previously described in Romano et al. (2007). Briefly, *C. burnetii* clone 4, phase II, strain Nine Mile bacteria were provided by Ted Hackstadt (Rocky Mountain Laboratories, NIAID, NIH, Hamilton, MT) and handled in a biosafety level II facility. For initial infection, 80% confluent Vero cells were infected with *C. burnetii* for 2 weeks at 37°C under 5% CO2. *C. burnetii* were obtained from infected cells by lysis of the host cells followed a differential centrifugation of the supernatants. Purified *C. burnetii* in the resulting pellet were resuspended in PBS and frozen at −70°C.

### Infection of cells with *C. burnetii*

Cells were plated on coverslips distributed in 6 or 24 well-plates and 0.5–1 ml of a dilution of *C. burnetii* suspension was added per well. Afterwards the cells were incubated at 37°C in an atmosphere of 95% air and 5% CO2 for the indicated time periods as described (Campoy et al., 2013).

### Ectopic expression and confocal microscopy

GFP-LC3, YFP-Gal8, YFP-Gal3, YFP-Gal9, YFP-Gal10, YFP-Gal4, YFP-Gal, YFP-Gal8 (R275H), YFP-Gal8 (R69H), and GFP-Gal3 (R186S) or RFP-NDP52 transfected CHO cells were analyzed by confocal microscopy using an Olympus FluoViewTM FV1000 confocal microscope (Olympus, Argentina), with the FV10-ASW (version 01.07.00.16) software. Images were processed using Adobe CS3 (Adobe Systems). Confocal images (0.39-μm sections) were collected.

### Super-resolution microscopy

High-resolution images were acquired on a ElyraPS1 microscope system (Zeiss, Carl Zeiss-Strasse 22 73447 Oberkochen, Germany) using a 100 oil-immersion lens (NA 1.46). This SIM system can achieve a resolution of 100 nm along the x–y axis and 300 nm along the z-axis. Laser lines at 488 and 561 nm were used for excitation. SIM images (15 images with five different phases for 3 different angular orientations of illumination for each SIM image) were acquired with an EMCCD camera (Andor Technology Ltd., Millennium WaySpringvale Business Park, Belfast BT12 7AL, UK; 1,002 1,004 pixels) and processed with Zen (Zeiss) software.

### Video-microscopy

Some *in vivo* experiments were recorded using an inverted AxioObserver Z1 microscope fitted with a Zeiss Axiocam® MRm camera (Carl Zeiss, Oberkochen, Germany) and operated by Axiovision® software. Images were acquired with a Plan-Apo 100 × /1.46 oil immersion objective (Carl Zeiss, Oberkochen, Germany). For video microscopy, the fluorophore excitation system was composed of a Colibri system (Zeiss, Carl Zeiss, Oberkochen, Germany) with 365, 470, 555, and 590 nm LEDs. For pH measurements, images were adquired in a LEICA fluorescence microscope spinning disk (AF6000-X; Campus CNRS CCHB, Bio Imaging Center Lille—Campus Lille 1, Université de Lille).

### Indirect immunofluorescence

The cells grown on coverslips were fixed with 3% paraformaldehyde solution in PBS for 10 min at room temperature, washed with PBS followed by quenching with 50 mM NH4Cl in PBS. Subsequently, cells were permeabilized with 1% saponin in PBS containing 1% BSA, and further incubated with the primary antibody dilution in PBS. Afterwards, the coverslips were incubated with a conjugated secondary antibody (Jackson immune Research Laboratories, EE. UU). After 3x washing with PBS cells were mounted with Mowiol (plus Hoechst) and examined by confocal microscopy as described previously (Campoy et al., 2013).

### Microinjection of infected CHO cells

Infected CHO cells were injected as previously described (Teitelbaum et al., 1999) with 3 kDa Texas red dextran, using a Micromanipulator 5,171 and a Transjector 5,426 plus (Eppendorf). Needles were pulled on a Sutter pipette puller, model P97. Infected CHO cells were microinjected with an initial pressure of 600 k Pa. Successive optical sections were collected and analyzed in a spin disk microscope (Olympus). The z series collection was initiated once diffusion of the injected marker was determined by imaging fluorescence. Injected cells were imaged for 20 min immediately after microinjection.

### pHrodo coupled to *S. aureus* assay

Preparations of *Staphylococcus aureus* particles conjugated to pHrodo were performed as previously described (Kobayashi et al., 2010). Heat-dead bacteria were washed twice and resuspended in PBS at an appropriate concentration (1:10 or 1:20 dilution). pHrodo-succinimidyl ester (Invitrogen, P36600) was added to the bacteria suspension and the samples were incubated in buffer bicarbonate for 45 min at room temperature. After conjugation, bacteria were washed and finally suspended in PBS. The resulting suspension (~10 μl) was mixed with 90 μl of culture medium and added to cells by replacing the medium. After incubation with the mixture for 1 h at 37°C, cells were washed with fresh medium to remove particles or not phagocytosed bacteria, and cells were subsequently incubated for 2 h.

### Beclin 1 knockdown

A double-hit protocol was performed as previously described in Vázquez and Colombo (2010). HeLa cells were plated in six-well-plates at 40% confluence and then transfected with GFP (control) or GFP and Beclin 1-KD (plasmid) After 24 h, cells were transfected with 24 h later, cells were transfected again with the GFP (control) or GFP and Beclin 1-KD (plasmid) again. Twenty four hours later cells were infected with *C. burnetii* during 48 h.

### Bacterial viability and replication

A fluorescent infectious FFU assay was used to quantify the replication and viability of *C. burnetii* in MEF wt or Atg5−/− cells or in Beclin1 KD HeLa cells. In brief, the infected cells were lysed and samples were serially diluted. Vero cells were infected with these lysates in a 24 well-plate. After 72 h of infection, Vero cells were fixed and processed for fluorescent microscopy. Approximately 1,000 cells were scored per coverslip to determine an average number of FFU for each sample.

### pH measurements

For standard curves, *C. burnetii* infected cells were incubated with 10 mM nigericin and 10 mM valinomycin in the pH 7.0 calibration Buffer. The coverslips were subsequently incubated for 10 min in pH calibration buffers adjusted to pH of 4.0, 5.0, 6.0, or 7.0. Then, they were incubated with Oregon green dextran 10,000 MW at a concentration of 1 mg/ml in culture medium overnight. Images for ratiometric calculations were acquired in a Leica spinning disk at excitation wavelengths of 440 nm and 480 nm with a fixed emission wavelength of 520 nm. Ratio values for the standard curves were determined by measuring the average 480/440 ratio value. To determine the pH of *C. burnetii*'s vacuoles, infected cells were incubated with Oregon green dextran in culture medium overnight. Ratio values were plotted against the standard curves to determine the pH of *C. burnetii* replicative vacuoles.

### Coating of beads with beta-lactamase

Amine functionalized beads were incubated for 4 h in 8% glutaraldehyde in PBS on a rolling wheel at RT. Activated beads were washed and resuspended in lactamase containing solutions in saturating conditions (estimated following the manufacturer's instructions). They were left overnight on a rolling wheel at 4°C. Beads were then washed and stocked at 4°C in PBS.

### CCF4 assay for *Coxiella* vacuole rupture

CHO cells were seeded on coverslips in 24-well plates at 2 × 10^4^ cells per well for experiments with fixed samples. CHO cells were infected with *C. burnetii* and after 48 h, they were loaded with 0.1 mM CCF4/AM substrate (Invitrogen) in EM buffer (120 mM NaCl, 7 mM KCl, 1.8 mM, 0.8 mM MgCl2, 5 mM glucose and 25 mM Hepes, pH 7.3) containing 6 mM probenecid, an inhibitor of anions transporters, for 2 h at RT, washed twice with EM and transferred to fresh medium containing 6 mM probenecid for a further 30 min. CCF4-loaded CHO cells were directly incubated with beta-lactamase coated beads (see Coating of beads with lactamase) for 8 h at 37°C. The adquisition was performed with a spinning-disk confocal microscope is composed of two diode laser sources emitting at wavelength of 488 and 440 nm (Omicron, Germany) injected in a spinning-disk confocal system (Yokogawa CSU-X1, Tokyo, Japan) adapted on a Leica (Lognes, FR) DMI6000B inverted microscope. Cells were imaged using a 63 × water-immersion objective (Leica HCX Plan Apo NA 1.20 W Corr CS). Fluorescence emitted was then successively routed by a dichroic mirror (Semrock Di01-T405/488/568/647 and 405/440/514 respectivlely), spectrally filtered (Semrock, FF01-520/35 nm) and detected with a Quatem 512SC EMCCD coupled to an optical zoom (×2). Every 512 × 512 image was acquired using 300 ms exposure time. The acquisition was performed by separated spectra to avoid potential overlap between emissions. Results were analyzed using ImageJ software (live cell imaging).

## Author contributions

MM and AB Methodology and Investigation. MM, FL, and MC Writing—Review and Editing. FL and MC Funding Acquisition, Resources, Supervision.

### Conflict of interest statement

The authors declare that the research was conducted in the absence of any commercial or financial relationships that could be construed as a potential conflict of interest.
