# Supplementary material for: Alterations of the Coxiella burnetii Replicative Vacuole Membrane Integrity and Interplay with the Autophagy Pathway
Source: Front Cell Infect Microbiol. 2017 Apr 24;7:112. doi: 10.3389/fcimb.2017.00112 (PMC5401879; doi:10.3389/fcimb.2017.00112)
Supplement: Supplementary file 1 [file DataSheet1.PDF]

Supplemental Information

Figure S1

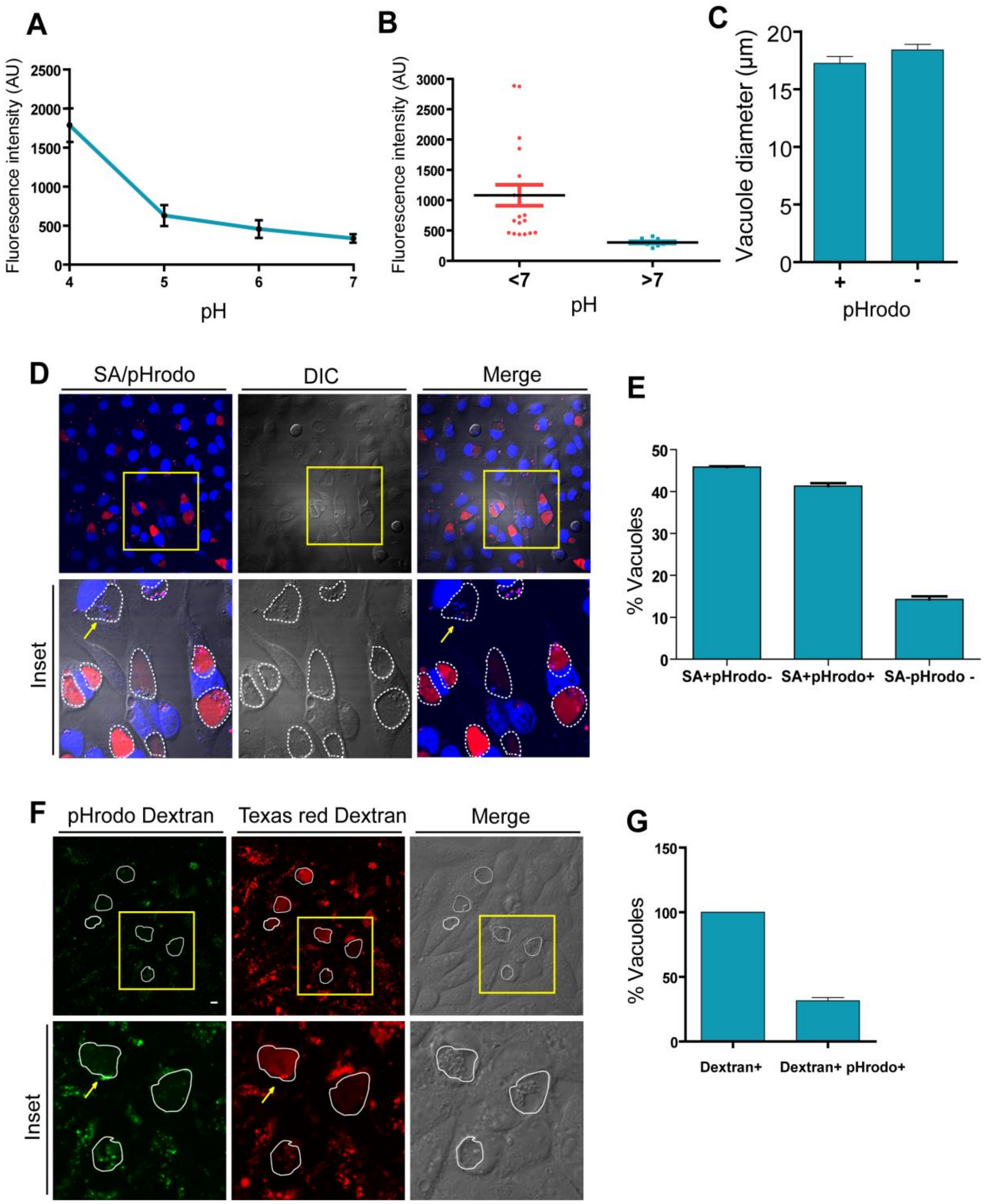

Figure S2

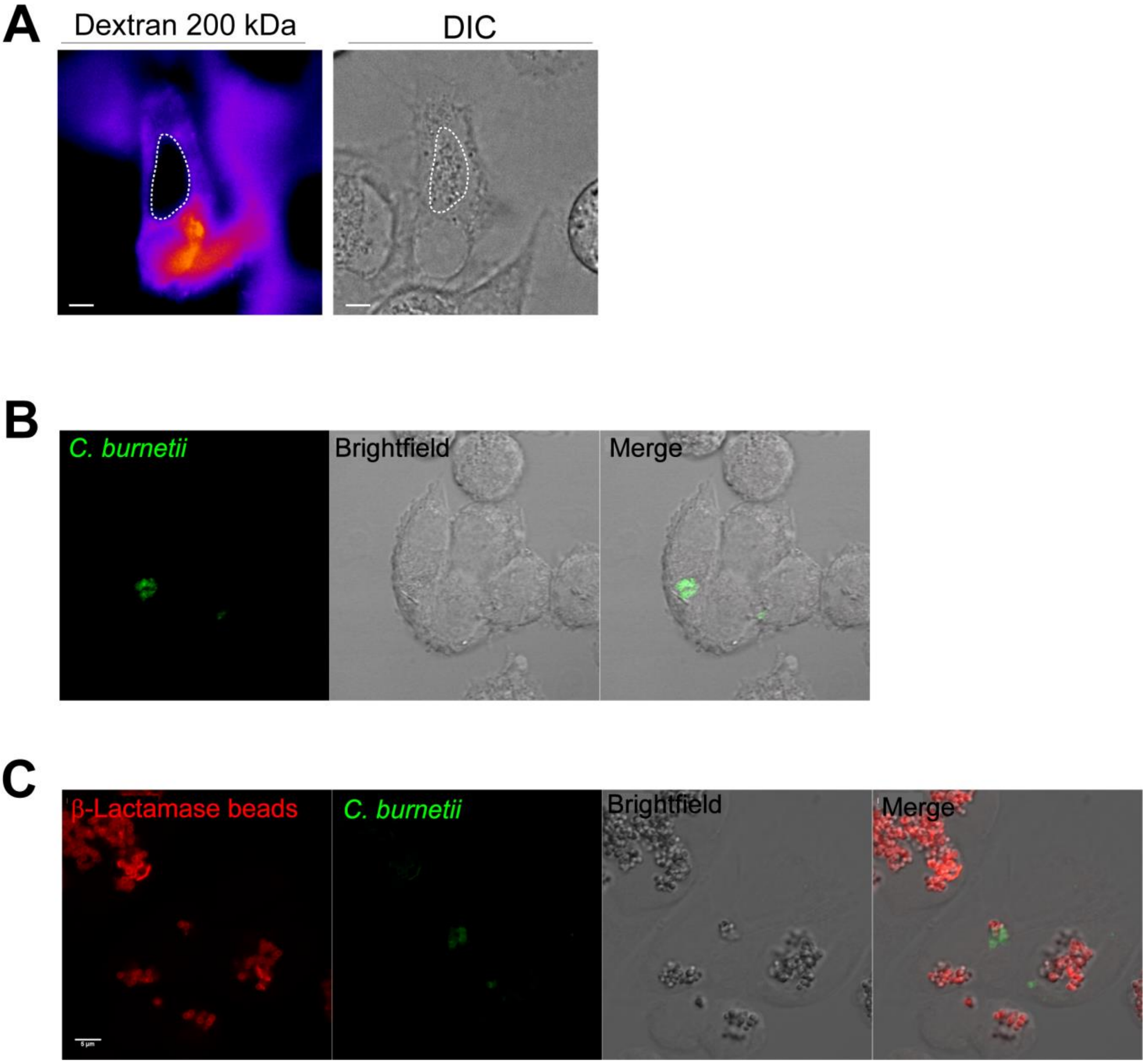
A
Dextran 200 kDa
DIC
B
C. burnetii
Brightfield
Merge
C
β-Lactamase beads
C. burnetii
Brightfield
Merge

Figure S3

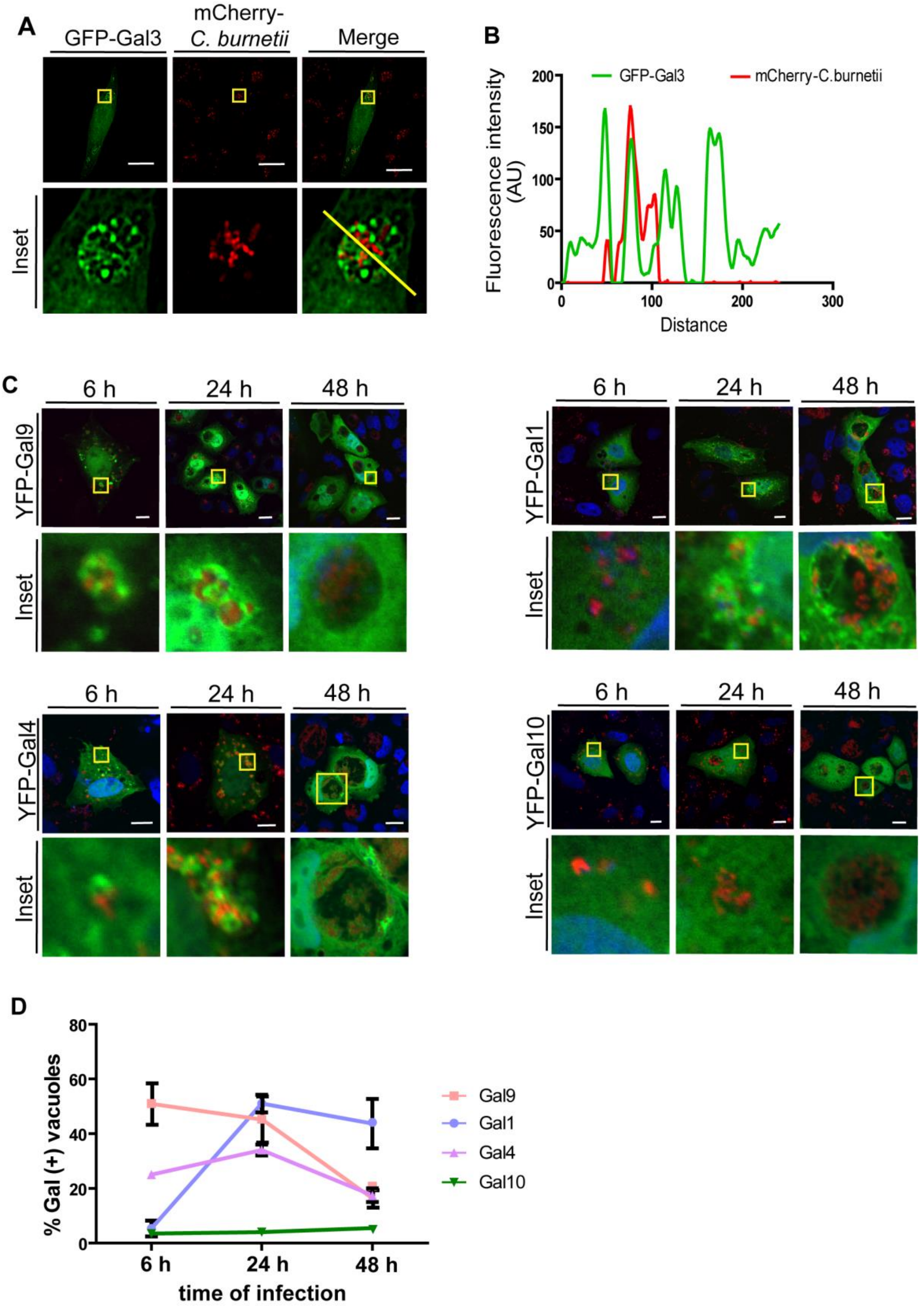

Figure S4

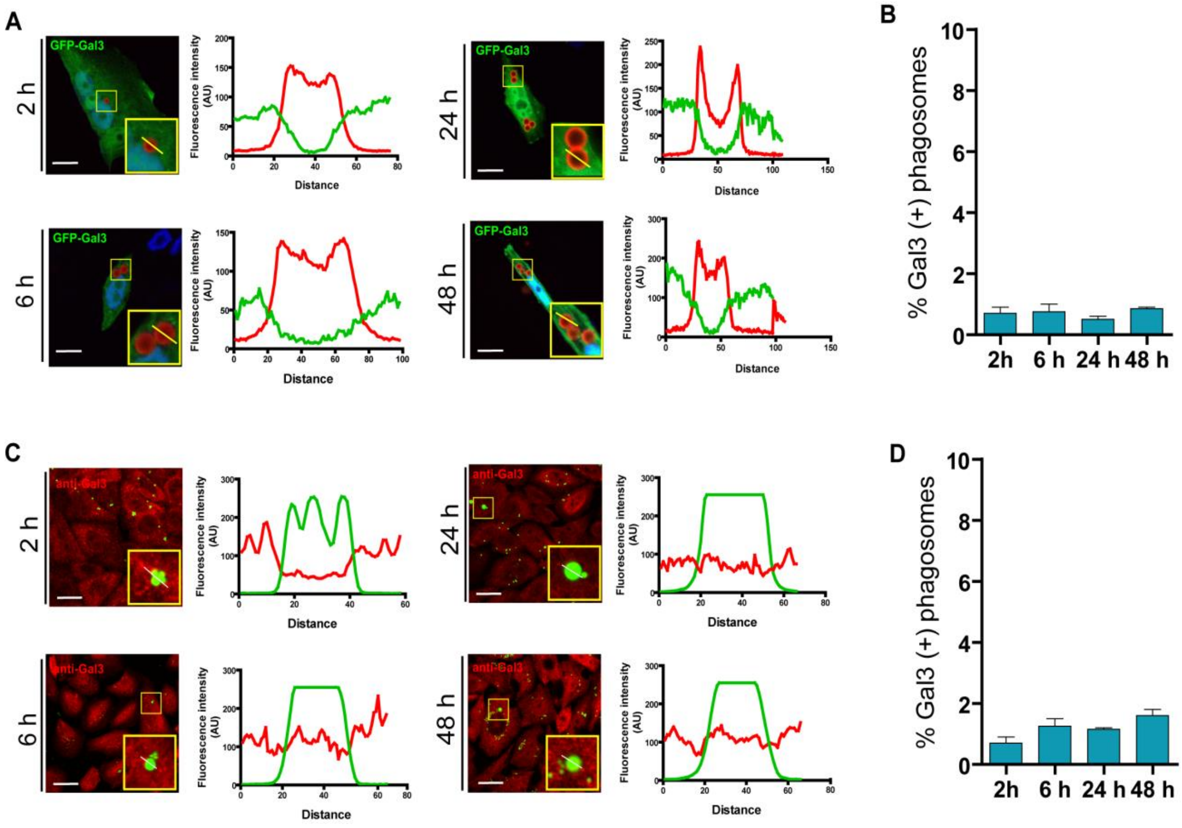
A
2 h
6 h
24 h
48 h
GFP-Gal3
Fluorescence intensity (AU)
Distance
B
% Gal3 (+) phagosomes
2h
6 h
24 h
48 h
C
anti-Gal3
D

Figure S5

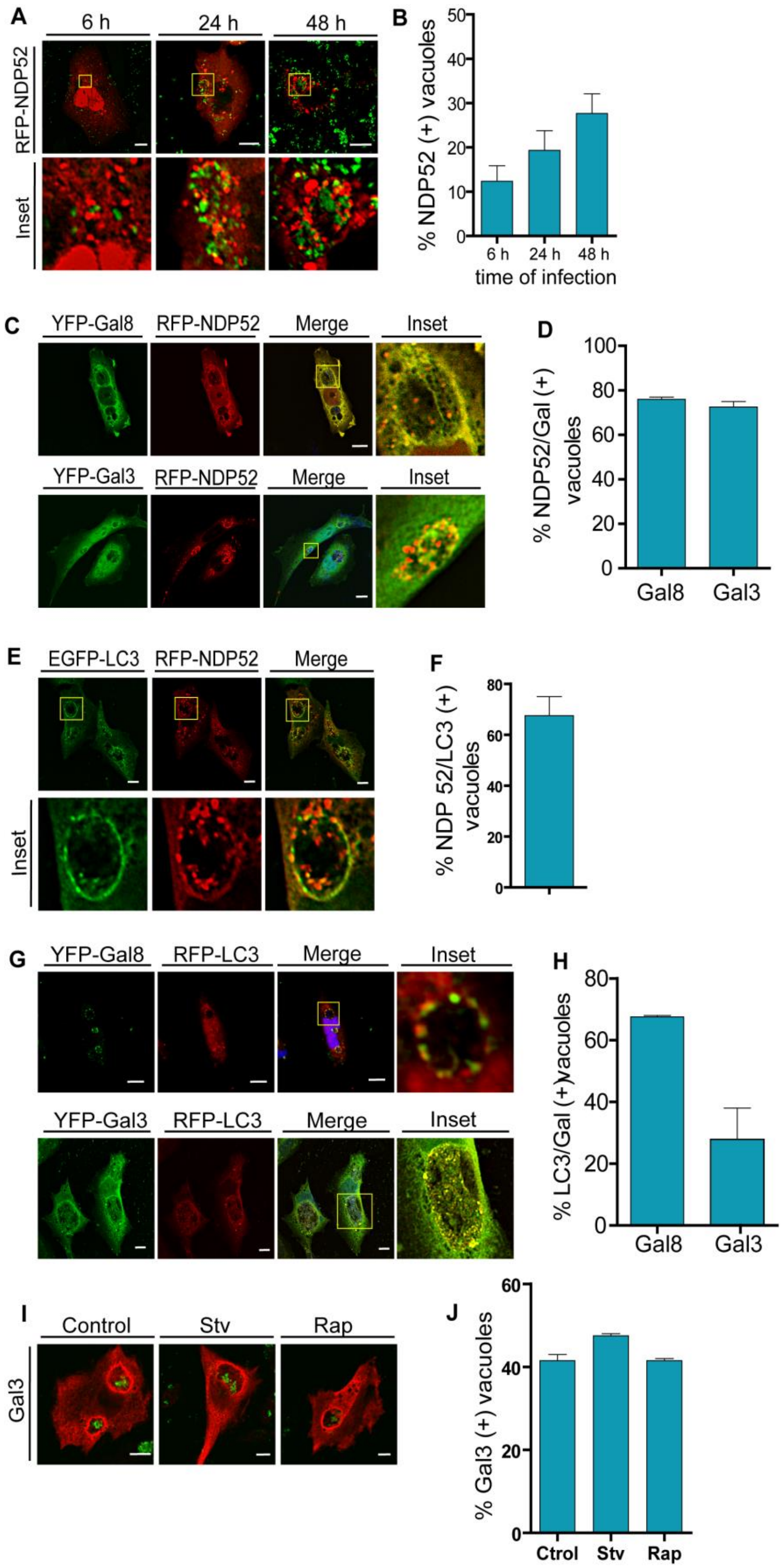

A
6 h
24 h
48 h
RFP-NDP52
Inset
B
% NDP52 (+) vacuoles
6 h
24 h
48 h
time of infection
C
YFP-Gal8
RFP-NDP52
Merge
Inset
YFP-Gal3
RFP-NDP52
Merge
Inset
D
% NDP52/Gal (+) vacuoles
Gal8
Gal3
E
EGFP-LC3
RFP-NDP52
Merge
Inset
F
% NDP 52/LC3 (+) vacuoles
G
YFP-Gal8
RFP-LC3
Merge
Inset
YFP-Gal3
RFP-LC3
Merge
Inset
H
% LC3/Gal (+) vacuoles
Gal8
Gal3
I
Control
Stv
Rap
Gal3
J
% Gal3 (+) vacuoles
Ctrol
Stv
Rap

Figure S6

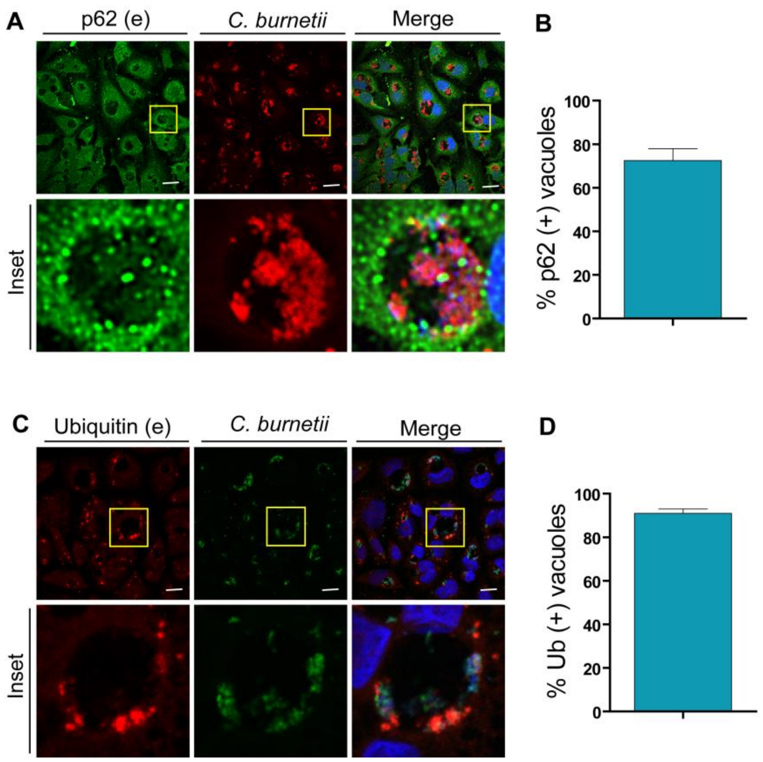

Figure S1: Labeling with pHrodo indicates heterogeneity of the pH of the CRVs.

(A). Fluorescence intensity standard curve of beads coupled to pHrodo incubated at different pH solutions. (B). Quantification of fluorescence intensity of vacuoles incubated with dead *S. aureus* coupled with pHrodo and divided in two groups (pH under or above 7). (C). Quantification of the vacuole diameter in pHrodo positive or negative. (D). CHO cells were infected with *C. burnetii*. After 48h, cells were incubated pHrodo Texas red coupled to dead *S. aureus* (Hoechst), and analyzed by confocal videomicroscopy. (E). Quantification of the percentage of vacuoles *S. aureus* positive (Hoechst) and pHrodo positive (red) or negative (black). Yellow arrows indicate a vacuole with *S. aureus* (Hoechst) and no pHrodo fluorescence. (F). CHO cells were infected with *C. burnetii.* After 48 h, cells were incubated with Texas red dextran (red) and pHrodo dextran (green) and analyzed by confocal videomicroscopy. (G). Quantification of the percentage of vacuoles Texas red dextran positive (red) and both Texas red dextran and pHrodo dextran positive vacuoles (red

and green). Yellow arrows indicate a vacuole with Texas red fluorescence and no pHrodo fluorescence. Scale bar: 10 µm.

Figure S2: Microinjection and CCF4-AM/β-lactamase assay.

(A). CHO cells were infected with *C.burnetii* for 48 hours and subsequently microinjected with 200 kDa green-tagged dextran particles. Cells were immediately visualized by confocal microscopy. (B). CHO cells were infected with GFP-*C. burnetii* for 48 h. Afterwards, cells were imaged by fluorescence wide field confocal microscopy. (C). CHO cells were infected with GFP-*C. burnetii* for 48 h. Afterwards, cells were allowed to internalize 1 µm latex beads coupled with β-lactamase and imaged by fluorescence wide field confocal microscopy. Scale bar: 5 µm.

Figure S3: Galectins decorate the CRV membrane.

(A). CHO cells were transiently transfected with GFP-Gal3 and infected with mCherry-*C. burnetii*. At 48 h of infection, cells were fixed and analyzed using SIM imaging. (B). Fluorescence intensity profile along the yellow line depicted in the inset of panel D. (C). CHO cells were transiently transfected with YFP-Gal1, -Gal9, -Gal4 or -Gal10 and then infected with mCherry-*C. burnetii*. At 6 h, 24 h and 48 h of infection, cells were fixed and analyzed by confocal microscopy. (D). Quantification of the percentage of the CRVs labeled with the different galectins from images like the ones depicted in panel B. The data represent the mean ± S.E.M. of at least three independent experiments (n>50 cells/group). Scale bar: 10 µm.

Figure S4: Galectin 3 is not recruited to beads-containing phagosomes.

(A). CHO cells were transiently transfected with GFP-Gal3 and then incubated with 3 µm rhodamine beads. At 2 h, 6 h, 24 h and 48 h of incubation, cells were fixed and analyzed by confocal microscopy. Right panels: fluorescent intensity profile along the yellow line in the inset of the corresponding image shown in the left panel. (B). Quantification of the percentage of

phagosomes labeled with GFP-Gal3 at different incubation times. (C). CHO cells were incubated with FITC-beads. At 2 h, 6 h, 24 h and 48 h of incubation, cells were fixed and subjected to immunofluorescence against Gal3. Then, cells were analyzed by confocal microscopy. Right panels: fluorescent intensity profile along the yellow line in the inset of the corresponding image shown in the left panel. (D). Quantification of the percentage of phagosomes labeled with Gal3 at different incubation times. Scale bar: 10 µm.

Figure S5. The adaptor protein NDP52 and LC3 associate to the *C. burnetii*-containing compartment labeled with Gal3.

(A). CHO cells were transfected with RFP-NDP52 and at 24 h post transfection were infected for 6 h, 24 h and 48 h with *C. burnetii*. The cells were fixed and subjected to indirect immunofluorescence using an antibody against C. burnetii (green) and visualized by confocal microscopy. (B). Quantification of the percentage of CRVs labeled with NDP52 from images like the ones depicted in panel A. (C). CHO cells were co-transfected with YFP-Gal8 (upper panels) or YFP-Gal3 (lower panels) and pRFP-NDP52. At 24 h post-transfection, cells were infected with *C. burnetii* for 48 h. Cells were fixed and examined by confocal microscopy. Insets show the recruitment of galectins (green) and NDP52 (red) at the limiting membrane and inside of the *C. burnetii*-containing vacuoles (D). Quantification of the percentage of NDP52-positive vacuoles that recruit Gal8 or Gal3 from images as the ones shown in C. (E). CHO cells overexpressing pEGFP-LC3 were transfected with pRFP-NDP52. At 24 h post-transfection, cells were infected with *C. burnetii* for 48 h. Cells were fixed and examined by confocal microscopy. Insets show the recruitment of LC3 (green) and NDP52 (red) at the membrane of the vacuoles containing *Coxiella* (blue). (F). Quantification of the percentage of NDP52-positive vacuoles that recruit LC3. (G). CHO cells were co-transfected with YFP-Gal8 or YFP-Gal3 and RFP-LC3. After 24 h post-transfection, cells were infected with *C. burnetii* for 48 h. Cells were fixed, subjected to indirect immunofluorescence with specific antibodies against *C. burnetii* and examined by confocal

microscopy. Insets show the recruitment of galectins (green) and LC3 (red) at the limiting membrane of the vacuoles containing *Coxiella* (blue). (H). Percentage of LC3-positive vacuoles recruiting Gal8 or Gal3. The data represent the mean± S.E.M. of at least three independent experiments (n>50 cells/group). (I). CHO cells overexpressing Gal3 (red) were infected with *C. burnetii* for 48 h and subsequently incubated for 2 h in full nutrient medium (Control) or starvation medium in the absence (Stv) or in the presence of 50 ng/µl rapamycin (Rap). Cells were subjected to immunofluorescence with specific antibodies against Gal3 (red) and *C. burnetii* (green). (J). Quantification of the percentage of CRVs labeled with Gal3 for each condition depicted in panel I. The data represent the mean ± S.E.M. of at least two independent experiments (n>50 cells/group). Scale bar: 10 µm.

Figure S6: The adaptor protein p62 and ubiquitin are recruited to *C. burnetii*-containing vacuole.

(A). CHO cells were infected with *C. burnetii* for 48 h, fixed and subjected to indirect immunofluorescence using specific antibodies against p62 (green) and *C. burnetii* (red). (B). Quantification of the percentage of CRVs labeled with p62 (green) from images like the ones depicted in panel A. (C). CHO cells were infected with *C. burnetii* for 48 h, fixed and subjected to indirect immunofluorescence using specific antibodies against ubiquitin (red) and *C. burnetii* (green). (D). Quantification of the percentage of CRVs labeled with ubiquitin (red) from images like the ones depicted in panel C. The data represent the mean ± S.E.M. of at least three independent experiments (n>50 cells/group). Scale bar: 10 µm
